# Regulation of MORC-1 is key to the CSR-1–mediated germline gene licensing mechanism in *C. elegans*

**DOI:** 10.1126/sciadv.ado4170

**Published:** 2025-06-20

**Authors:** Jessica A. Kirshner, Colette L. Picard, Natasha E. Weiser, Nicita Mehta, Suhua Feng, Victoria N. Murphy, Anna Vakhnovetsky, Amelia F. Alessi, Connie Xiao, Kai Inoki, Sonia El Mouridi, Christian Frøkjær-Jensen, Steven E. Jacobsen, John K. Kim

**Affiliations:** ^1^ Department of Biology, The Johns Hopkins University, Baltimore, MD, USA.; ^2^ Department of Molecular Cell and Developmental Biology, University of California, Los Angeles, Los Angeles, CA, USA.; ^3^ Department of Pathology, Stanford University School of Medicine, Stanford, CA, USA.; ^4^ King Abdullah University of Science and Technology, Biological and Environmental Epigenetics Program, Thuwal, Saudi Arabia.; ^5^ Howard Hughes Medical Institute, University of California, Los Angeles, Los Angeles, CA, USA.

## Abstract

The Argonaute CSR-1 is essential for germline development in *C. elegans*. Loss of CSR-1 leads to the down-regulation of thousands of germline-expressed genes, supporting a model in which CSR-1 “licenses” gene expression via a poorly understood mechanism. In contrast, a small subset of genes is up-regulated in *csr-1* mutants, including *morc-1*, which encodes a conserved GHKL-type ATPase. We show that *morc-1* is overexpressed in *csr-1* mutants and accumulates over CSR-1 licensed targets, coinciding with aberrant gain of H3K9me3, reduced H3K36me3, and transcriptional repression. Notably, loss of *morc-1* fully rescues these chromatin defects and partially restores gene expression and fertility in *csr-1* mutants. Conversely, ectopic overexpression of MORC-1 in the wild-type germ line is sufficient to repress CSR-1 licensed targets and severely compromise fertility. These findings support a model in which CSR-1 prevents MORC-1 overexpression and consequent misregulation of CSR-1 licensed genes.

## Teaser

MORC-1 acts downstream of CSR-1 to regulate germline chromatin states and is a key component of the gene licensing mechanism.

## INTRODUCTION

Maintaining genome integrity is essential in the germ line, where gene expression must be tightly and precisely regulated. In *Caenorhabditis elegans*, this regulation is orchestrated by an integrated network of small RNA pathways that ensures that germline genes are expressed at appropriate levels, while non-germline genes and foreign genetic elements are stably silenced. A key component of this system is the small RNA-dependent surveillance machinery, which targets transposons and other deleterious elements through proteins in the highly conserved Argonaute family (*1*, *2*). However, the Argonaute protein CSR-1 functions differently from its counterparts: It binds a distinct set of endogenous small interfering RNAs (endo-siRNAs; 22G-RNAs) complementary to approximately 4000 endogenous protein-coding genes (PCGs). Rather than repressing these targets, CSR-1 is thought to promote—or “license”—their expression (*3*).

This licensing model is supported by the observation that loss of *csr-1* leads to modest down-regulation of most CSR-1 target genes, which comprise most genes expressed in the germ line (*3*, *4*). In addition to the global down-regulation of germline-expressed genes, *csr-1* mutants exhibit complete sterility and a range of other phenotypes, including meiotic nondisjunction, enlarged and disorganized P granules, aberrant expression of sperm-specific mRNAs in the hermaphrodite germ line, and global depletion of core histone proteins (*2*–*7*). Despite further characterization of the CSR-1 gene licensing pathway (*8*–*12*), the molecular mechanisms by which CSR-1 promotes expression of its targets remain largely unknown. Moreover, the sterility of *csr-1* mutants has posed a substantial challenge to dissecting this pathway in vivo.

In addition to its gene licensing function, CSR-1 has endonucleolytic “slicing” activity (*13*, *14*), a canonical feature of many Argonaute proteins. While the vast majority of CSR-1 targets are down-regulated in *csr-1* mutants, a small subset—approximately 100 genes—appears to be silenced by CSR-1, potentially by this target cleavage mechanism (*14*). The silencing function of CSR-1 is further supported by interactions with regulatory partners such as the Pumilio/FBF (PUF) protein FBF-1 and the translation elongation factor EFT-3, which together can inhibit translation elongation and reinforce silencing at specific loci (*15*, *16*). Among the silenced targets of CSR-1 is *morc-1*, which encodes the sole *C. elegans* homolog of the conserved *Microrchidia* (MORC) family of gyrase, heat-shock protein 90, histidine kinase, MutL (GHKL)-type ATPases (*14*, *15*).

MORC proteins are broadly conserved across plants and animals, with diverse functions including repressing transcription and maintaining repressive chromatin states (*17*–*27*). The specific mechanisms by which MORCs repress transcription vary. Some mammalian MORCs are thought to regulate H3K9me3 via interactions with the SETDB1/HUSH complex (*20*, *25*), while several *Arabidopsis* MORCs promote the establishment of DNA methylation (*24*). Alternatively, MORCs may recruit histone deacetylases (HDACs) to maintain repressive chromatin (*23*, *26*, *27*). In *C. elegans*, MORC-1 contributes to silencing by preventing euchromatin from spreading into repressive H3K9me3-enriched regions (*21*) and can compact chromatin in vitro in a DNA sequence–independent manner (*22*). Despite these roles, the in vivo targets and molecular mechanisms of MORC-1 action in *C. elegans* remain largely uncharacterized. Here, we show that targeted silencing of *morc-1* by CSR-1 is a substantial contributor to the CSR-1–mediated germline gene licensing mechanism. Moreover, MORC-1 overexpression alone is sufficient to partially recapitulate key phenotypes observed in *csr-1* mutants, including down-regulation of CSR-1 targets and sterility.

## RESULTS

### *morc-1(−)* partially rescues *csr-1* sterility

**Fig. 1.**
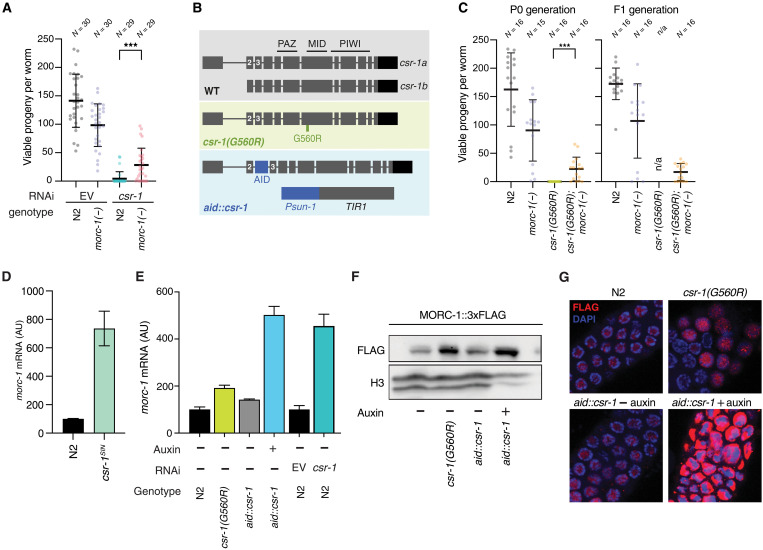
*morc-1(−)* is a suppressor of *csr-1*. (**A**) Fertility of wild-type (WT; N2) or *morc-1(−)* worms grown on either EV or *csr-1* RNAi. Each point represents the viable progeny produced by an individual worm. (**B**) Diagram of CSR-1 gene and protein structure for both isoforms, as well as the two *csr-1* mutants generated in this study: *aid::csr-1* and *csr-1(G560R)*. Exons 2 and 3 are labeled for reference. The approximate location of the three protein domains PAZ, MID, and PIWI is also shown. (**C**) Fertility of wild-type, *morc-1(−)*, *csr-1(G560R)*, and *csr-1(G560R); morc-1(−)* double mutant worms. Each point represents the viable progeny produced by an individual worm. Left shows progeny of the first generation (P0) grown at the *csr-1(G560R)* nonpermissive temperature of 25°C, while the right shows progeny of second generation (F1) worms, also grown at 25°C. Because *csr-1(G560R)* P0 worms did not produce any progeny at 25°C, their fertility in the F1 generation could not be assayed (n/a, not applicable). (**D**) Up-regulation of *morc-1* mRNA in the *csr-1*^SIN^ mutant by qPCR. Error bars represent SD between two technical replicates. (**E**) Up-regulation of *morc-1* mRNA in wild type (N2), *csr-1(G560R)*, and *aid::csr-1*, as well as on *csr-1* or EV RNAi, by qPCR. Error bars represent SD between two technical replicates. AU, arbitrary units. (**F**) Western blot of MORC-1::3xFlag protein in both *csr-1(G560R)* and *aid::csr-1*, with H3 as a loading control. (**G**) Immunofluorescence of MORC-1::3xFlag (red) in *csr-1(G560R)* and *aid::csr-1* in dissected germ lines of indicated genotype and treatment. DNA was stained with 4′,6-diamidino-2-phenylindole (DAPI). [(D) to (F)] Worms were treated with either 0 μM [(−) auxin] or 100 μM auxin [(+) auxin]. [(A) and (C)] ***P < 0.001, one-tailed *t* test.

*C. elegans* lacking *csr-1* exhibit severe germline defects and are sterile, and the few embryos that do form arrest by the 100-cell stage (*3*). We hypothesized that this phenotype may result in part from the aberrant overexpression of one or more genes normally silenced by CSR-1. Among the ~100 such genes (*14*), we focused on *morc-1*, which we previously found to be essential for germline development and fertility in worms, consistent with conserved roles for MORC proteins across species (*18*, *28*). To test for a genetic interaction between *csr-1* and *morc-1*, we assessed the fertility of wild-type and *morc-1(−)* animals subjected to either control [empty vector (EV)] or *csr-1* RNA interference (RNAi). As expected, wild-type animals were completely sterile on *csr-1* RNAi. In contrast, *morc-1(−)* animals retained partial fertility on *csr-1* RNAi (Fig. 1A), suggesting that loss of *morc-1* can partially suppress the sterility associated with *csr-1* depletion.

To further investigate the interaction between *morc-1* and *csr-1*, we used CRISPR-Cas9 to generate two new, viable *csr-1* alleles (Fig. 1B). The first, *csr-1(G560R)*, harbors a point mutation homologous to the antimorph mutation in the Argonaute ALG-1, which disrupts microRNA passenger strand removal (*29*) and is predicted to impair small RNA binding. CSR-1(G560R) is well expressed and localizes properly to perinuclear granules, similar to wild-type CSR-1 (fig. S1, A to C). The second allele, *aid::csr-1*, incorporates an auxin-inducible degron (AID) tag between the second and third exons of *csr-1*, allowing for germline-specific degradation of CSR-1 upon auxin treatment via the TIR1 F-box protein (*30*), which we express under a germline-specific promoter (Fig. 1B and fig. S1D). Both alleles affect both the *csr-1a* and *csr-1b* isoforms (Fig. 1B).

We validated these strains as functional *csr-1* mutants by assaying fertility and gene expression. At 25°C, *csr-1(G560R)* animals were sterile, and fertility was similarly abolished in *aid::csr-1* animals across an auxin concentration gradient (fig. S1E). Both mutants displayed hallmark CSR-1 gene licensing defects, with mild down-regulation of CSR-1 licensed targets (*4*) and up-regulation of CSR-1–silenced targets as previously defined (fig. S1F) (*14*). Consistent with reduced CSR-1 protein levels in *aid::csr-1* and defective small RNA loading in *csr-1(G560R)*, CSR-1–bound 22G-RNAs were moderately destabilized in both mutants, with a more pronounced effect in *aid::csr-1* (fig. S1G), likely reflecting partial retention of small RNA binding in *csr-1(G560R)*.

To better understand the effect of *csr-1(G560R)* on small RNA loading, we further analyzed the 22G-RNA binding capacity of CSR-1(G560R) using RNA immunoprecipitation sequencing (RIP-seq) of GFP::3xFlag-tagged wild-type CSR-1 and mutant CSR-1(G560R). Although both immunoprecipitates (IPs) were enriched for 22-nt small RNAs (fig. S2A), CSR-1(G560R) bound a smaller proportion of 22-nt small RNAs compared to wild-type CSR-1 (fig. S2A). As expected, both wild-type CSR-1 and CSR-1(G560R) IPs were enriched for 22-nt small RNAs corresponding to CSR-1 targets and depleted for small RNAs corresponding to MUT-16 targets (fig. S2, B to D). However, this enrichment was reduced in the CSR-1(G560R) samples. Moreover, the small RNAs bound by CSR-1(G560R) were skewed toward the 3′ ends of target genes, including *morc-1* (fig. S2, C and D). CSR-1(G560R) also recovered proportionally more MUT-16–dependent 22G-RNAs (fig. S2, B to D), although it remains unclear whether this reflects a true shift or simply a relative increase due to recovering fewer CSR-1–dependent 22G-RNAs. Overall, these data indicate that *csr-1(G560R)* is a hypomorphic allele with reduced capacity to bind its cognate 22G-RNAs. We conclude that both *csr-1(G560R)* and *aid::csr-1* represent valid *csr-1* loss-of-function mutants that phenocopy the sterility and gene expression defects observed by *csr-1* RNAi and in the *csr-1* null mutants.

We next compared the fertility of *csr-1(G560R)* animals to that of the *csr-1(G560R); morc-1(−)* double mutant and found that *morc-1* loss also partially rescues the sterility defect of *csr-1(G560R)* (Fig 1C and fig. S3). Notably, this rescue was stable across generations, as fertility in the double mutant was maintained transgenerationally (Fig. 1C). These findings support the conclusion that *morc-1* functions as a genetic suppressor of *csr-1.*

### MORC-1 is overexpressed in *csr-1* mutants

We next investigated the molecular basis of *csr-1* rescue by *morc-1* loss. A previous study by Gerson-Gurwitz *et al.* (*14*) identified 133 putative slicing targets of CSR-1 that are up-regulated in a slicing-inactive *csr-1* mutant—hereafter referred to as “CSR-1–silenced targets”—one of which is *morc-1*. In addition to being a potential CSR-1 slicing target, the *morc-1* transcript may be translationally repressed by the CSR-1–FBF-1–EFT-3 ternary complex (*15*, *16*). To test whether *morc-1* is repressed by CSR-1, we measured its expression at both the mRNA and protein levels in multiple *csr-1* mutant backgrounds. Using quantitative polymerase chain reaction (qPCR), we confirmed that *morc-1* transcript levels are up-regulated more than sixfold in the slicing-inactive *csr-1* mutant (*csr-1*^SIN^) (Fig. 1D), consistent with previous findings (*14*)*.* We further examined *morc-1* expression in our newly generated *csr-1* mutants and found that, relative to wild-type animals, *morc-1* is up-regulated at both the mRNA and protein levels (Fig. 1, E to G). A similar increase was observed upon *csr-1* RNAi in wild-type worms (Fig. 1E and fig. S4, A to D). Together, these results confirm that *morc-1* is up-regulated in the absence of functional CSR-1, either through direct loss of slicing activity or through an indirect mechanism that remains to be defined.

### MORC-1 binds the transcriptional start sites of germline-expressed genes and spreads in *csr-1* mutants

**Fig. 2.**
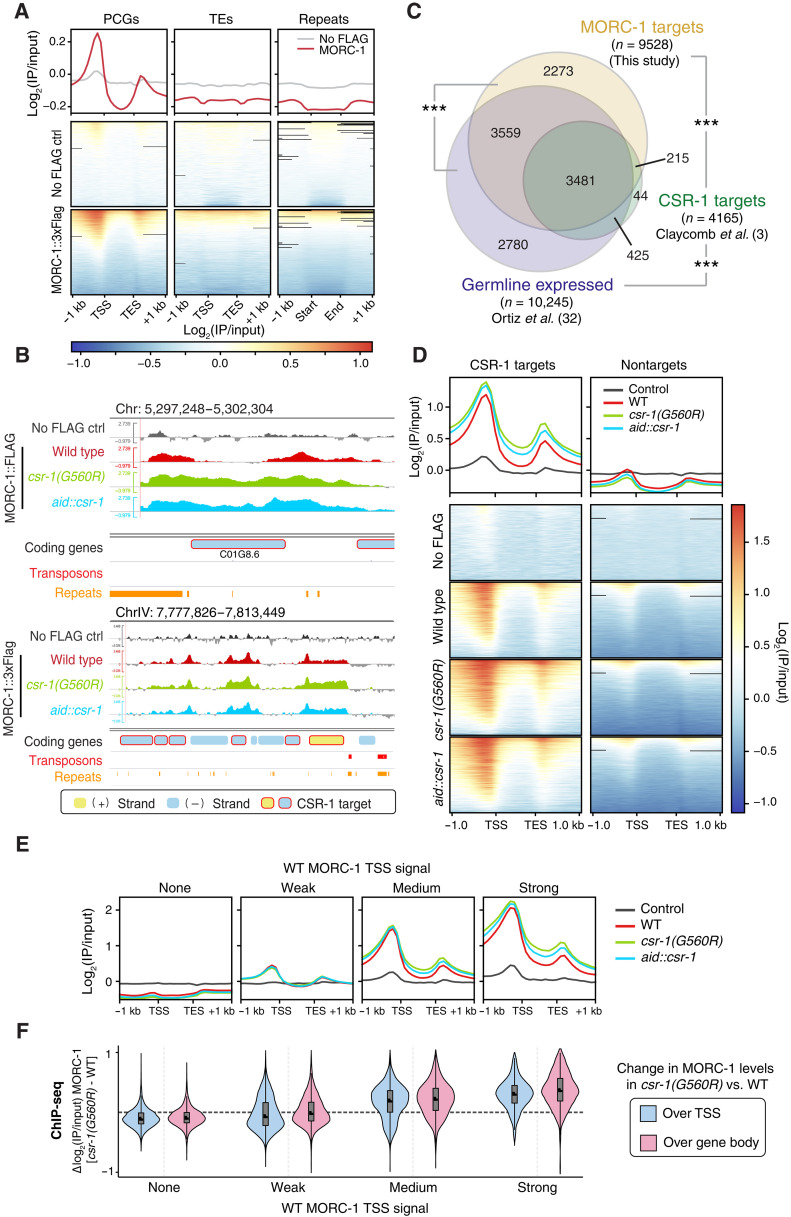
MORC-1 binds at the transcriptional start site of protein coding genes and spreads in *csr-1* mutants. (**A**) Metaplots and heatmaps of anti-Flag ChIP-seq signal from purified germline nuclei of the MORC-1::3xFlag expressing strain or control wild-type worms lacking Flag (no-Flag control). Metaplots show average signal across all protein-coding genes (PCGs), transposon elements (TEs), and repeat regions (repeats). In heatmaps, each row corresponds to a single PCG, TE, or repeat. Features were scaled to 1-kb length. Each IP sample was normalized to matched input sample [log_2_(IP/input)], and values represent average of two replicates (averaged before log transformation). (**B**) Example genome browser images showing average log_2_(IP/input) MORC-1::3xFlag signal in wild-type germ line (red), *csr-1(G560R)* (green), and *aid::csr-1* (blue), as well a no-Flag control (gray). Genes, transposons, and repeats shown on bottom tracks. Genes on the forward and reverse strands are colored yellow and blue, respectively, while CSR-1 targets are circled red. (**C**) Overlap between CSR-1 targets (green) (*3*), germline expressed genes (purple) (*32*), and MORC-1 targets identified in this study based on MORC-1 enrichment over the promoter and transcriptional start site (TSS) (yellow). ***P ~ 0, hypergeometric test. (**D**) Metaplots and heatmaps showing MORC-1::3xFlag localization in wild type and both *csr-1(G560R)* and *aid::csr-1* [(+) auxin], over CSR-1 target genes versus nontargets. (**E**) Average germline log_2_(IP/input) MORC-1::3xFlag signal in wild type (red), *csr-1(G560R)* (green), *aid::csr-1* (blue), and no-Flag control (gray), over genes binned by MORC-1 TSS signal in wild type. Genes were scaled to 1-kb length. Plots are over genes binned by promoter (±500 bp around TSS) MORC-1 levels (see data S2). (**F**) Distribution of change in average MORC-1::3xFlag ChIP-seq signal in *csr-1(G560R)* compared to wild type, over either the TSS region (±500 bp around TSS, blue) or gene body (pink), in genes binned based on MORC-1 TSS signal in wild type [bins same as (E)].

*C. elegans* MORC-1 is a highly conserved DNA binding protein capable of condensing DNA and chromatin in vitro (*22*), but its function in vivo remains poorly defined. To investigate the consequences of MORC-1 overexpression in a *csr-1* mutant background, we first characterized its chromatin binding profile in wild-type animals. We performed chromatin immunoprecipitation sequencing (ChIP-seq) in purified germline nuclei from a strain in which MORC-1 was endogenously tagged with a 3xFlag epitope via CRISPR-Cas9 (see Materials and Methods) (*31*). Unexpectedly, despite the conserved role of MORCs in silencing repetitive elements, *C. elegans* MORC-1 was depleted from transposable elements (TEs) and repetitive sequences in the germline genome (Fig. 2, A and B, and fig. S5, A to C). Instead, MORC-1 bound specifically and robustly to the promoter region near the transcriptional start sites (TSSs) of PCGs (Fig. 2, A and B, and fig. S5, A to D), with binding intensity correlating with germline gene expression levels (fig. S5, B to D). Accordingly, CSR-1 targets—which are typically highly expressed in the germ line (fig. S6A)—as well as other germline-enriched genes (*32*), were strongly bound by MORC-1 (Fig. 2, B to D, and fig. S6B). This trend held regardless of the specific dataset used to define CSR-1 targets (fig. S6C). Given that these datasets largely overlap (fig. S6D), we focused subsequent analyses on the Claycomb *et al.* (*3*) list of CSR-1 targets, which is widely used in the field.

The genes with the highest promoter MORC-1 occupancy in wild-type animals—including many CSR-1 targets—show mild up-regulation in a previously published *morc-1(−)* RNA sequencing (RNA-seq) dataset (fig. S7, A and B) (*21*). Moreover, genes up-regulated in *morc-1(−)* are also significantly enriched for the active chromatin mark H3K36me3 and depleted of the repressive mark H3K9me3 (fig. S7C), consistent with MORC-1 acting primarily at actively transcribed loci. Together, these results suggest that despite binding primarily to the promoters of highly expressed genes, MORC-1 may function to temper their expression, revealing a potentially repressive role for MORC-1 at germline TSSs.

We next performed ChIP-seq for MORC-1::3xFlag in the germ lines of our newly generated *csr-1* mutant strains, where MORC-1 is overexpressed (Fig. 1, D to F). In wild-type animals, MORC-1 binding was largely restricted to TSSs; however, in both *csr-1* mutants, MORC-1 occupancy increased further at target gene promoters and spread into the gene bodies, where it is normally absent (Fig. 2, B and D, and fig. S8, A and B). This overaccumulation occurred most prominently at genes already strongly bound by MORC-1 in wild-type animals and was not observed at non–MORC-1 targets (Fig. 2, E and F, and fig. S8, A and B). Furthermore, the gain of MORC-1 signal within gene bodies strongly correlated with increased binding at corresponding TSSs in the *csr-1* mutant background (Fig. 2, E and F, and fig. S8, B and C). Therefore, CSR-1 targets and other genes strongly bound by MORC-1 in wild type became further enriched for MORC-1 across both the TSS and gene body regions in *csr-1* mutants. In contrast, nontarget genes, TEs, and repetitive regions remained depleted for MORC-1 binding in *csr-1* mutants (Fig. 2, B and D to F, and fig. S8, A to C). These findings suggest that loss of CSR-1 promotes aberrant overexpression and consequent spreading of MORC-1 across actively transcribed germline genes.

### MORC-1 spreading in *csr-1* mutants represses gene expression

**Fig. 3.**
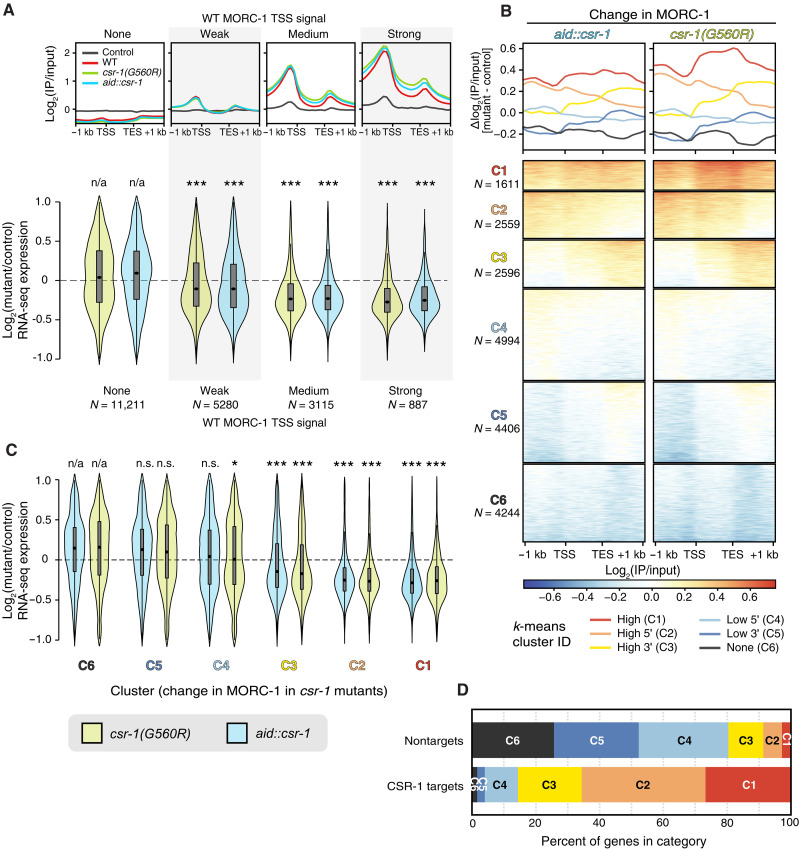
Genes highly bound by MORC-1 are consistently down-regulated in both *csr-1(G560R)* and *aid::csr-1*. (**A**) Top: Metaplots of MORC-1::3xFlag ChIP-seq signal in wild type, *csr-1(G560R)*, and *aid::csr-1* [(+) auxin] over genes binned by wild-type MORC-1 signal over TSS (same as Fig. 2E). Bottom: Distribution of RNA-seq log_2_ fold change values in indicated mutant over control, estimated by DESeq2 (*65*), across genes binned by wild-type MORC-1 level at TSS as in (A). A small number of genes outside of *y* in [−1,1] not shown. (**B**) Metaplots and heatmaps of change in MORC-1::3xFlag ChIP-seq signal in both *csr-1(G560R)* and *aid::csr-1* [(+) auxin] compared to control [difference in log_2_(IP/input) signal] over all PCGs (rows of heatmap). Genes were clustered using the *k*-means algorithm into six clusters, named C1 to C6. (**C**) Distribution of RNA-seq log_2_ fold change values in indicated mutant over control, estimated by DESeq2, across gene clusters from (C). (**D**) Percent of genes in each cluster from (C) that are CSR-1 targets (*3*) versus nontargets. (B and D) A small number of genes with *y* outside [−1,1] not shown. Significance testing: ***P < 0.0001, and*P < 0.01 and *P* > 0.001. n.s., not significant; two-sample Wilcoxon rank-sum test with the null hypothesis that the observed distribution of log_2_ fold change values is drawn from the same distribution as (B) genes lacking MORC-1 at the TSS (none) or (D) genes in cluster 6 (“C6”). [(B) to (D)] Control for *csr-1(G560R)* is N2 (wild type), and control for *aid::csr-1* [(+) auxin] is *aid::csr-1* [(−) auxin].

We hypothesized that the ectopic spreading of MORC-1 across CSR-1 targets contributes to their down-regulation in *csr-1* mutants. Consistent with this, genes that are highly bound by MORC-1 in wild type—and that gain the most MORC-1 in *csr-1* mutants (Fig. 2E)—were significantly down-regulated in both the *csr-1(G560R)* and *aid::csr-1* mutant strains (Fig. 3A). In contrast, genes with little or no MORC-1 binding remained largely unaffected (Fig. 3A).

To further explore this relationship, we performed *k-*means clustering of genes based on change in MORC-1 occupancy across both *csr-1* mutants (Fig. 3B). The resulting clusters showed consistent patterns between the two *csr-1* mutant strains. One cluster of genes (C1) displayed strong aberrant MORC-1 gain across both the promoter and the entire gene body relative to wild type, while others showed MORC-1 accumulation predominantly at the 5′ end (C2) or 3′ end (C3). Genes in clusters C4 and C5 exhibited minimal MORC-1 changes, while genes in C6 showed a loss of MORC-1 binding. Notably, genes in clusters C1 and C2—those with strong MORC-1 gain across either their entire length or in their promoter/5′ end—were the most strongly down-regulated in both of our *csr-1* mutant strains (Fig. 3C). Genes in C3, which gained MORC-1 primarily over their 3′ ends, were also down-regulated, although less so than C1 and C2, suggesting that MORC-1 accumulation at the promoter or 5′ region has a more potent repressive effect. In contrast, genes in clusters with minimal or no MORC-1 gain relative to wild type were not significantly down-regulated.

A regression analysis further supported a modest but consistent correlation between ectopic MORC-1 gain within gene bodies and gene down-regulation across both *csr-1* mutant strains (fig. S9). The genes in clusters C1 and C2, and to a lesser extent C3, were highly expressed in the germ line (fig. S10, A and B) and include nearly all CSR-1 targets (Fig. 3D). Together, these findings suggest that ectopic overaccumulation of MORC-1, particularly at promoters and 5′ regions, contributes to the down-regulation of germline-expressed genes, including CSR-1 targets, in the absence of CSR-1—highlighting a previously unappreciated repressive role for MORC-1 at actively transcribed germline genes.

### *morc-1(−)* rescues *csr-1* defects in gene expression and chromatin states

**Fig. 4.**
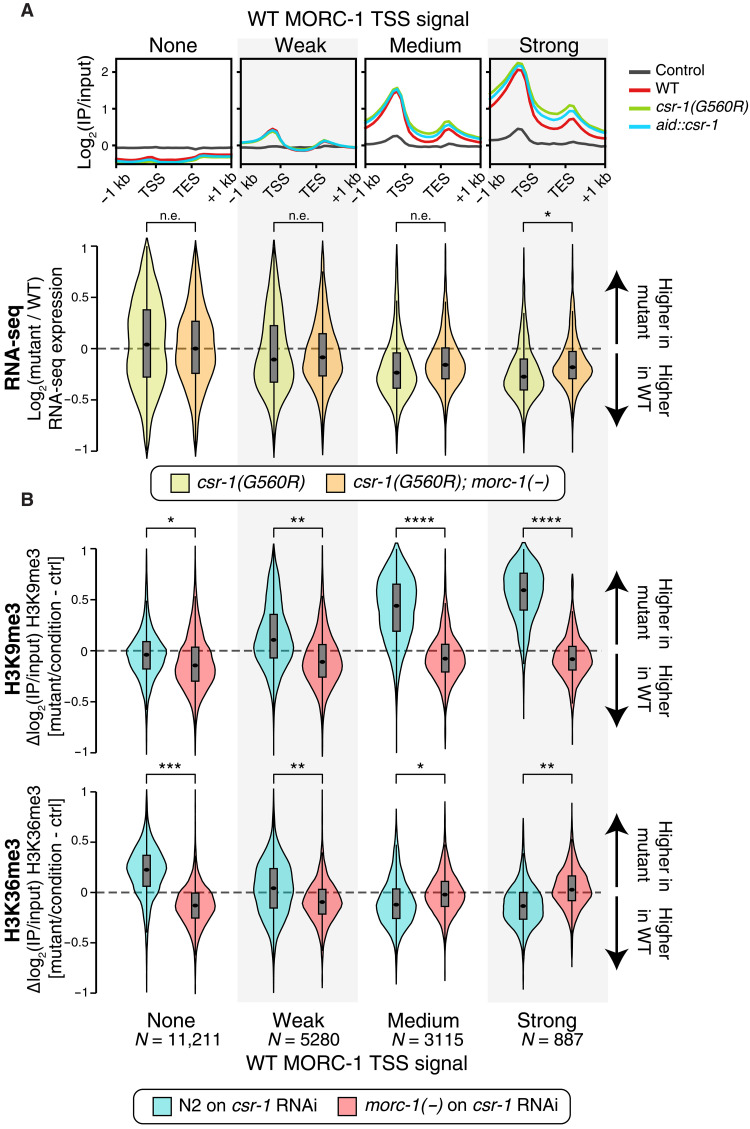
*morc-1(−)* rescues *csr-1* defects in gene expression and chromatin states. (**A**) Top: Same as Fig. 2E: average MORC-1::3xFlag ChIP-seq signal in wild type, *csr-1(G560R*, *aid::csr-1*, and no-Flag control, over genes binned by promoter (±500 bp around TSS) MORC-1 levels. Bottom: Distribution of change in gene expression in indicated mutant versus wild type, over genes binned based on MORC-1 levels at the TSS in wild type, as in the top panel. Log_2_(fold change) values were estimated by DESeq2 (*65*). Number of genes in each bin shown at the bottom (B). (**B**) Difference in H3K9me3 and H3K36me3 ChIP-seq signal [log_2_(IP/input)] in indicated mutant and/or condition, compared to wild-type worms treated with control RNAi. Genes were again binned on the basis of MORC-1 levels at the TSS in wild type, as in (A) and (B). [(A) and (B)]Stars indicate effect size as measured using Cohen’s *d*. n.e., no/minimal effect (|*d*| < 0.2); *|*d*| > 0.2, **|*d*| > 0.5, ***|*d*| > 0.9, and ****|*d*| > 1.5. All comparisons give *P* ~ 0 by Student’s *t* test due to large sample size.

If the overaccumulation and spreading of MORC-1 at CSR-1 target and other germline-expressed genes underlies their down-regulation in *csr-1* mutants, then loss of *morc-1* should alleviate these expression defects. To test this, we performed RNA-seq in *csr-1(G560R); morc-1(−)* animals*.* As expected, the down-regulation of genes that strongly gained MORC-1 binding in *csr-1(G560R)*—including many CSR-1 targets—was partially rescued by *morc-1* loss, while genes not bound by MORC-1 were unaffected (Fig. 4A and fig. S11, A and B). In contrast, expression of CSR-1–silenced targets (*14*) was not rescued by loss of *morc-1* (fig. S11B). Instead, these silenced targets were even further up-regulated in the *csr-1(G560R); morc-1(−)* double mutant. Most CSR-1–silenced targets also gained substantial MORC-1 binding in our *csr-1* mutants (fig. S11C), suggesting that their enhanced up-regulation in the double mutant likely reflects the combined loss of both MORC-1–mediated transcriptional repression and CSR-1–mediated posttranscriptional repression. Together, these data suggest that loss of *morc-1* partially rescues gene expression defects in *csr-1(G560R)* by reversing the repressive effects of ectopic MORC-1 overaccumulation at CSR-1–licensed genes.

Given that *csr-1* mutants also exhibit altered chromatin states (*4*, *33*), we next asked whether these chromatin defects might be driven by MORC-1 overexpression. As previously reported (*21*), *morc-1(−)* mutants alone showed only mild global reductions in H3K9me3 and modest gains in H3K36me3 (fig. S12A). In contrast, *csr-1* RNAi in wild-type worms resulted in a marked gain of H3K9me3 across more than 2000 1-kb bins genome-wide and particularly within the bodies of PCGs (fig. S12, B and C), consistent with prior observations of increased H3K9me3 over CSR-1 target genes in *csr-1* mutants (*33*). Under wild-type conditions (control RNAi), these regions were normally strongly depleted of H3K9me3. In addition, *csr-1* depletion caused loss of the active mark H3K36me3 in ~900 1-kb bins, which also mapped primarily to gene bodies and strongly overlapped with regions that gained H3K9me3 (figs. S12, B and C, and S13A). These data suggest that repressive H3K9me3 spreads inappropriately into actively transcribed gene bodies in the absence of CSR-1, while active chromatin features such as H3K36me3 are concurrently lost—indicative of the disrupted chromatin boundaries previously reported in *csr-1* mutants (*4*, *33*).

We hypothesized that these chromatin changes in *csr-1* RNAi conditions are a consequence of ectopic MORC-1 overaccumulation and spreading. Supporting this, genes that were highly bound by MORC-1 in wild-type germ lines—and showed the strongest MORC-1 gain in *csr-1* mutants (Fig. 2E)—also exhibited the greatest gain of H3K9me3 and loss of H3K36me3 following *csr-1* RNAi [Fig. 4B (blue) and figs. S13, A to C, and S14, A to C]. This chromatin defect was fully rescued in *morc-1(−)* mutants subjected to *csr-1* RNAi [Fig. 4B (red) and figs. S13, A to C, S14, A to C, and S15A], while *csr-1* RNAi did not rescue the mild chromatin defects of *morc-1* mutants (fig. S15, B and C). These findings suggest that MORC-1 overaccumulation in *csr-1* mutants drives the inappropriate deposition of H3K9me3 and loss of H3K36me3 over highly expressed germline genes, including CSR-1 targets, revealing a key role for MORC-1 in shaping chromatin states downstream of CSR-1.

### MORC-1 overexpression is sufficient to mimic *csr-1–*dependent germline gene licensing

Our findings support a model in which MORC-1 normally binds at the promoters of CSR-1 targets and other germline-expressed genes in wild-type animals but overaccumulates at these loci in *csr-1* mutants, leading to chromatin defects that contribute to transcriptional repression and sterility. To test whether elevated levels of MORC-1 are sufficient to recapitulate these *csr-1* defects, we attempted to overexpress MORC-1 in the germ line using a transgene-based approach in wild-type worms. However, this experiment is technically challenging, as MORC-1 promotes transgene silencing in *C. elegans* (*17*), potentially silencing its own overexpression construct. Moreover, if MORC-1 overexpression is toxic—like *csr-1* loss—then we could fail to recover viable transgenic lines. Multiple strategies to generate MORC-1–overexpressing animals were initially unsuccessful (table S1).

**Fig. 5.**
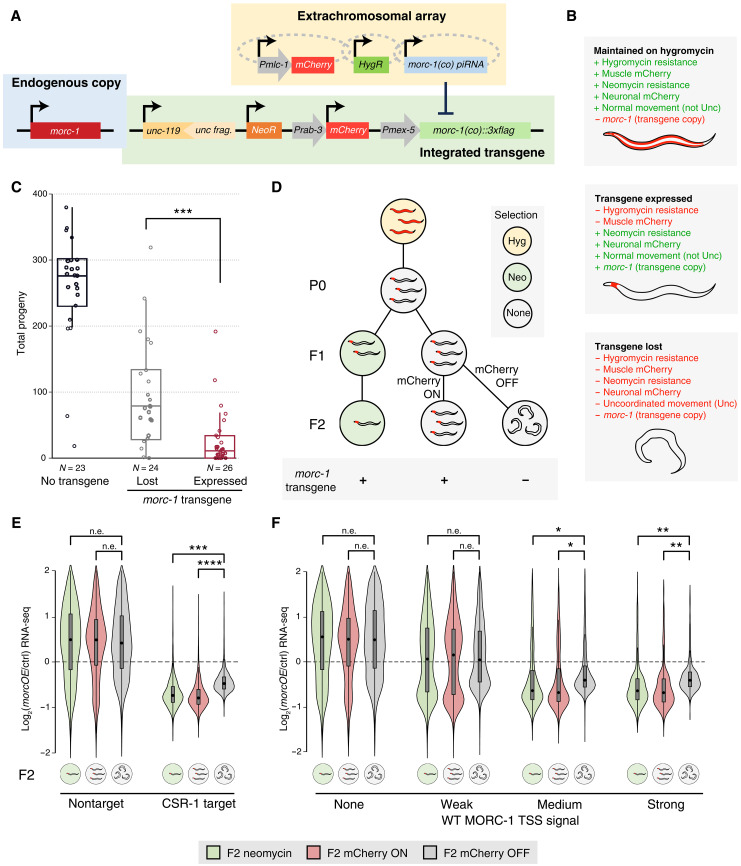
MORC-1 overexpression in wild-type germline phenocopies *csr-1* fertility and expression defects. (**A**) Schematic of the conditional germline MORC-1 overexpression line (*morcOE*; see Materials and Methods). (**B**) Expected phenotypes of *morcOE* stock worms maintained on hygromycin (extrachromosomal array retained), worms removed from hygromycin that have lost the extrachromosomal array and express all genes on the integrated transgene, and worms that have lost the integrated transgene. (**C**) Fertility of F2 *morcOE* worms off selection, comparing worms that have lost the integrated *morc-1* transgene to those that retained it. The transgene was considered lost if either mCherry expression was lost or worms became uncoordinated [Unc; see (B) and data S4]. ***P < 0.001, two-tailed *t* test. (**D**) Schematic of experimental design (see Materials and Methods). F2 worms not on neomycin were manually separated into transgene-expressing and transgene-lost populations based on expression of neuronal mCherry. *morcOE* worms maintained on neomycin always retained mCherry expression but were sick and had low fertility, so few worms remained by F2. Hyg, hygromycin; Neo, neomycin; none, no selection. (**E**) Expression changes by RNA-seq of CSR-1 targets versus nontargets in the three populations of *morcOE* F2 worms assayed: neomycin selection, no selection with mCherry ON, and no selection with mCherry OFF, see (D), each compared to a no-transgene control. (**F**) Expression changes by RNA-seq of genes binned by wild-type MORC-1 occupancy at TSS (Fig. 2E), in the three populations of *morcOE* F2 worms assayed, compared to a no-transgene control. [(E) and (F)] Expression change shown is the log_2_(fold change) value estimated by DESeq2 (*65*). Effect size measured using Cohen’s *d*. n.e., no/minimal effect (|*d*| < 0.2); *|*d*| > 0.2, **|*d*| > 0.5, ***|*d*| > 0.9, and ****|*d*| > 1.5. All comparisons give *P* ~ 0 by Student’s *t* test, due to large sample size.

We ultimately succeeded in generating a strain (*morcOE*) that conditionally overexpresses *morc-1* in the germ line (see Materials and Methods). We used a MosTi-based safe harbor site (*34*) to insert a single-copy transgene containing codon-optimized *morc-1::3xflag* under a germline-specific promoter into the *unc-119* locus (Fig. 5A). This transgene also carried a neomycin resistance gene and a neuronal mCherry marker. To avoid early lethality from *morc-1* overexpression, we coexpressed an artificial piRNA (Piwi-interacting RNA) from an extrachromosomal array to silence the transgene through piRNA interference (piRNAi) (*35*). This array also included a muscle mCherry marker, a hygromycin resistance gene, and the CRISPR components (sgRNA and Cas9) used for *morc-1* transgene integration. To induce *morc-1* overexpression conditionally, worms were removed from hygromycin, enabling loss of the piRNA array and desilencing of the codon-optimized *morc-1* (Fig. 5B).

Following hygromycin removal, we observed frequent loss of the neuronal mCherry marker and reappearance of the uncoordinated (Unc) phenotype in subsequent generations, suggesting loss of the integrated transgene. Genotyping confirmed loss of the integrated *morc-1* transgene array in mCherry*-*negative worms but not in mCherry-positive worms (fig. S16, A to C), validating mCherry as a reliable proxy for transgene presence. Because the extrachromosomal array expresses Cas9 and an sgRNA targeting the integrated transgene array, we hypothesize that some transgenes were reexcised before array loss, resulting in heterozygous animals that could give rise to progeny lacking the transgene altogether—especially in the absence of neomycin selection. This occurred at high frequency, consistent with a deleterious effect of *morc-1* overexpression.

To assess the effect *of morc-*1 overexpression on fertility, we quantified brood size in three groups: control worms with only endogenous *morc-1::3xflag*, *morcOE* worms actively overexpressing *morc-1* (*morcOE*^+^), and *morcOE* siblings that had lost the transgene and no longer overexpressed *morc-1* (*morcOE*^−^; based on phenotypic indicators, see Fig. 5B). While all worms derived from the *morcOE* background showed reduced fertility compared to wild type, *morcOE*^−^ animals were significantly more fertile than their *morcOE*^+^ siblings (Fig. 5C), indicating that *morc-1* overexpression alone is sufficient to impair fertility.

We next examined gene expression by performing RNA-seq on *morcOE* worms across three generations: the first generation grown off hygromycin (P0), and two successive generations of their progeny (F1 and F2) (Fig. 5D). One population was maintained on neomycin to preserve the integrated transgene, while another was kept off selection to allow transgene loss. By the F2 generation, the nonselected population showed clear loss of transgene markers (Fig. 5, B and D, and fig. S16, A to C). We manually separated *morcOE^+^* and *morcOE^−^* individuals in the F2 using neuronal mCherry expression as a marker for transgene loss (Fig. 5D and fig. S16, A to C). While endogenous *morc-1* levels remained similar across all samples, the codon-optimized *morc-1* transgene was only detectable in *morcOE^+^* animals (fig. S17, A to C; see Supplementary Text). Consistent with our model, genes down-regulated in *morcOE^+^* worms were highly enriched for germline-expressed genes (fig. S18, A to C), while *morcOE^−^* worms showed few down-regulated genes with no specific tissue or Gene Ontology (GO) term enrichment (fig. S18, A to C). Moreover, CSR-1 targets, other germline-expressed genes, and genes highly bound by MORC-1 in the wild-type germ line were significantly down-regulated in *morcOE^+^* samples but not in *morcOE^−^* controls (Fig. 5, E and F, and fig. S19, A to C). Together, these findings demonstrate that germline overexpression of MORC-1 is sufficient to repress CSR-1 targets and other germline-expressed genes, phenocopying key aspects of *csr-1* mutant gene expression defects.

## DISCUSSION

The prevailing model of CSR-1–mediated gene licensing posits that CSR-1 directly promotes gene expression, although the underlying mechanisms remain largely uncharacterized (*3*, *4*). Our findings identify regulation of *morc-1* expression as a critical component of this licensing pathway, with MORC-1 acting downstream of CSR-1 to fine-tune levels of germline-expressed genes. In *csr-1* mutants, *morc-1* is overexpressed three- to fivefold [Fig. 1 and (*14*)], leading to its excessive accumulation at target genes (Fig. 2), where it promotes repressive chromatin and correlates with gene down-regulation (Figs. 3 and 4). MORC-1 is enriched at highly expressed genes—many of which are also CSR-1 targets in the germ line (*3*)—indicating that a major consequence of MORC-1 overexpression is repression of CSR-1–licensed genes. This suggests that one mechanism by which CSR-1 promotes germline gene expression is through repression of *morc-1*.

Loss of *morc-1* fully rescued the aberrant spread of repressive chromatin in *csr-1* mutants (Fig. 4), suggesting that ectopic MORC-1 overaccumulation is the primary driver of these chromatin defects. This is consistent with MORC-1’s known role as a chromatin regulator, both in *C. elegans* (*21*, *22*) and in other species (*17*, *18*, *23*–*27*). While repressive chromatin is expected to result in gene silencing—and we observe this—loss of *morc-1* only partially restores the gene expression defects of *csr-1* mutants. This implies that additional mechanisms downstream of CSR-1 contribute to gene regulation, beyond MORC-1–mediated repression. Several such mechanisms have been described. For example, CSR-1 binding to its target transcripts can prevent them from being silenced by the piRNA pathway (*9*–*11*) . Further, the association with CSR-1–class 22G-RNAs with CSR-1 may prevent them from being misloaded into the silencing Argonaute HRDE-1 (*12*), thus protecting transcripts from inappropriate silencing.

Because these pathways converge on CSR-1 targets—which comprise the vast majority of germline-expressed genes (*3*)—we postulate that the sterility observed in *csr-1* mutants stems from the widespread down-regulation of germline gene expression, driven by both MORC-1 overexpression and the failure of parallel licensing mechanisms downstream of CSR-1. This model is further supported by our observations that *morc-1* loss can partially suppress the sterility of *csr-1* mutants and that MORC-1 overexpression alone is sufficient to induce sterility and repress germline gene expression, including CSR-1 licensed targets. Thus, while multiple other pathways likely contribute to *csr-1* mutant defects, our work positions MORC-1 overexpression as a key effector through which CSR-1 regulates chromatin states and ultimately germline gene expression and fertility.

If CSR-1 does not always function directly to promote the expression of germline transcripts, then why are CSR-1–bound 22G-RNAs complementary to germline-expressed genes? A recent study offers an intriguing clue: CSR-1 is required to silence maternally deposited transcripts in the somatic blastomeres of early embryos (*36*). Because these cells inherit maternal germline transcripts, their timely clearance is essential for proper zygotic genome activation and somatic development. In this context, CSR-1 acts by slicing these maternally derived transcripts, thereby enabling the transition to zygotic transcription (*36*). This raises the intriguing possibility that in the maternal germ line, CSR-1 22G-RNAs target germline genes not solely to promote their expression but to mark them as “germline-lineage” transcripts. Following fertilization, this molecular memory could then be repurposed by CSR-1 to selectively cleave these transcripts in somatic cells, ensuring that germline-specific gene expression is appropriately silenced outside of the germ line. Further investigation is needed to clarify the function of CSR-1–bound 22G-RNAs and to understand how 22G-RNAs derived from germline transcripts are correctly sorted and loaded into CSR-1.

It remains unclear how *morc-1* becomes up-regulated in *csr-1* mutants. CSR-1 has canonical Argonaute slicing activity, guided by 22G-RNAs, and *morc-1* is among the CSR-1 “silenced” targets identified in the study of Gerson-Gurwitz *et al.* (*14*). Notably, these silenced targets—including *morc-1—*are much more strongly enriched for CSR-1–bound 22G RNAs than the “licensed” targets (fig. S2). This observation supports the hypothesis that CSR-1 directly silences *morc-1* through small RNA-guided slicing. However, we cannot rule out the possibility that *morc-1* up-regulation in *csr-1* mutants is an indirect effect. Additional studies aimed at dissecting this regulatory mechanism will be important for fully understanding CSR-1’s role in controlling *morc-1* expression and, more broadly, its function in gene regulation.

Our results also provide insights into the mechanism of action of MORC-1. ChIP-seq analysis revealed that MORC-1 is strongly enriched at gene promoters, particularly at those of highly expressed genes. This promoter-proximal localization aligns well with in vitro findings showing that MORC-1 binds DNA in a non–sequence-specific manner, prefers naked DNA, and is inhibited by competing nucleosomes (*22*). Accordingly, MORC-1 accumulates at regions of accessible chromatin, such as promoters of actively transcribed genes. This localization may be facilitated by its CW-type zinc finger domain, which in mammalian MORC3 and MORC4 recognizes the active promoter histone mark H3K4me3 (*37*–*39*). Although direct binding of *C. elegans* MORC-1 to H3K4me3 has yet to be reported, many key residues required for this interaction in mammalian MORCs are conserved in worms (*40*). Similar promoter-binding patterns have been observed for MORCs in plants and mammals, although their function at these sites remains unclear (*20*, *38*, *41*, *42*).

When overexpressed, MORC-1 accumulates further at its genomic targets in a manner proportional to its initial binding. This is consistent with in vitro data showing that MORC-1 can promote cooperative binding, with one molecule facilitating recruitment of additional MORC-1 molecules to adjacent DNA regions (*22*). We also previously demonstrated that DNA compaction scales linearly with MORC-1 concentration (*22*), suggesting that in *csr-1* mutants, elevated levels of MORC-1 at gene promoters and bodies could lead to increased chromatin compaction. This alone may be sufficient to reduce transcription by limiting the access of the transcriptional machinery to the DNA. In addition to compaction, MORC-1 may act through the recruitment of chromatin-modifying enzymes. For instance, human MORC2 has been shown to recruit H3K9me3 methyltransferases (*20*), and consistent with this, we observed elevated H3K9me3 at MORC-1 targets in *csr-1* mutants (Fig. 4B). MORCs in other organisms, including *Toxoplasma gondii*, have also been shown to recruit HDACs (*23*, *26*, *27*). We therefore hypothesize that MORC-1 represses gene expression through a combination of direct DNA compaction and recruitment of chromatin-modifying enzymes. Further studies will be necessary to dissect how MORC-1 contributes to histone modification changes and transcriptional repression.

Overexpression of MORCs is a hallmark of several cancers (*41*, *43*–*46*), while mutations in MORCs can affect fertility (*18*, *21*, *28*) and cause developmental disorders in humans (*20*). These findings underscore that precise MORC dosage is essential for proper gene expression and organismal development across diverse biological systems. Further investigation into the effects of MORC overexpression—particularly at gene promoters where its role remains poorly understood—will be critical to elucidate its broader regulatory functions.

In conclusion, our results suggest that tight regulation of MORC-1 is an integral part of the CSR-1 gene licensing mechanism, acting to down-regulate germline genes in *csr-1* mutants. The viability of germ cells requires proper control of MORC-1 levels, mediated in part through CSR-1–dependent silencing of *morc-1*.

## MATERIALS AND METHODS

### Experimental model and subject details

*C. elegans* strains used in this study are listed in Table 1. Strains were maintained using standard procedures (*47*) at 15° or 20°C unless indicated otherwise. In all cases, Bristol N2 strain was used as the wild-type control. Worms were fed OP50 *Escherichia coli* for all experiments except ChIP-seq of purified germline nuclei and those involving RNAi. Worms for ChIP-seq were fed HB101 *E. coli*, and worms for RNAi experiments were fed with HT115 *E. coli.*

**Table 1.** *C. elegans* strains used in this study. N2, wild-type strain Bristol N2; CGC, Caenorhabditis Genetics Center.

Genotype	Name used in manuscript	Source or reference	Identifier
Wild type, Bristol isolate		CGC	N2
*morc-1(tm6048)*	*morc-1(−)*	(*21*)	QK80
*csr-1(xk46[G560R]) IV*	*csr-1(G560R)*	This study	QK225
*aid::csr-1; sun-1p::tir1::mRuby*	*aid::csr-1*	This study	QK149
*csr-1(tor67[gfp::3xflag::csr-1]) IV*	*gfp::3xflag::csr-1(WT)*	(*71*)	JMC101
*csr-1(xk47[gfp::3xflag::csr-1(G560R)]) IV*	*gfp::3xflag::csr-1(G560R)*	This study	QK226
*csr-1(xk46[G560R]); morc-1(tm6048)*	*csr-1(G560R); morc-1(−)*	This study	QK227
*ltSi242[pOD1267/pAG31; Pcsr-1::csr-1(reencoded; D606A, D681A: isoform b numbering); cb-unc-119(+)]II; unc-119(ed3)III?; csr-1(tm892) IV/nT1[unc-?(n754)let-?](IV;V)*	*csr-1*^SIN^	(*14*)	OD1175
*morc-1::3xflag*		(*21*)	QK84
*morc-1::3xflag; csr-1(xk46[G560R])*		This study	QK228
*morc-1::3xflag; aid::csr-1(xk17); sun-1p::tir1::mRuby*	*morc-1::3xflag; aid::csr-1*	This study	QK229
kstSi107pSEM417 *(Pmex-5::morc-1(PATC)::3xFlag::gpd-2::ce-gfp*), pSEM371 (*unc-119* integration fragment), pGH8 (*Prab-3::mCherry*), pCFJ594 (NeoR)] III; kstEx75[pCFJ2474 (*Psmu-2::Cas9(PATC)::gpd-2::tagRFP(myr),* pSEM376 (sgRNA *ce-unc-119* locus), T1636 (*morc-1* recoded piRNAi), pSEM235 *(Pmlc-1::mCherry*), pCFJ782 (Hygro), 1-kb ladder	*morcOE*	This study	CFJ242

### Construction of transgenic animals

All CRISPR strains were generated according to the standard procedure, as described (*48*). The MORC-1 overexpression strain (*morcOE*) was created by first generating an extrachromosomal array by injecting into the *unc-119(ed3)* strain: (i) a piRNA that silences the recoded, codon-optimized *morc-1::3xflag* transgene (*morc-1(co)::3xflag*) in the germ line via the piRNA pathway (*35*); (ii) reagents to generate targeted array integrations (Cas9 and sgRNA); (iii) a Hygro^R^ transgene for selection; and (iv) *Pmlc-1::mCherry*. These initial transgenic worms were then injected with (i) the recoded *morc-1* transgene containing periodic A_n_/T_n_-clusters (PATCs) [*Pmex-5::morc-1(PATC)::3xFlag::gpd-2::ce-gfp*], (ii) 1-kb ladder DNA, (iii) *Prab-3::mCherry*, (iv) Neomycin^R^, and (v) a fragment for targeted array integration into the *unc-119(ed)* locus using the MosTi single-copy integration method (*34*). The full genotype of *morcOE* is kstSi107pSEM417 *(Pmex-5::morc-1(PATC)::3xFlag::gpd-2::ce-gfp*), pSEM371 (*unc-119* integration fragment), pGH8 (*Prab-3::mCherry*), pCFJ594 (NeoR)] III; kstEx75[pCFJ2474 (*Psmu-2::Cas9(PATC)::gpd-2::tagRFP(myr)*, pSEM376 (sgRNA *ce-unc-119* locus), T1636 (*morc-1* recoded piRNAi), pSEM235 *(Pmlc-1::mCherry*), and pCFJ782 (Hygro), 1-kb ladder.

### RNAi assays

Bacterial clones containing the RNAi of interest were grown from the Ahringer RNAi library (*49*) and administered by feeding, as reported previously (*50*). Cultures were inoculated from a single colony, grown for 12 to 16 hours in LB with carbenicillin (50 μg/ml), plated on isopropyl-β-D-thiogalactopyranoside–containing plates, and then induced for expression overnight at 25°C. Worms were plated as L1s after this induction period and grown at 20°C, unless indicated otherwise. In all cases, the EV L4440 was used as a negative control.

### Auxin assays

In experiments in which auxin (3-indoleacetic acid) (Sigma-Aldrich) was used, nematode growth media agar (NGM) plates were poured containing the indicated concentration of auxin. In ChIP-seq experiments in which worms were grown in liquid culture, the auxin was added directly to the cultures.

### Western blotting

For Western blots, worms were lysed in tris-glycine SDS sample buffer (Thermo Fisher Scientific), run on an 8 to 16% Novex WedgeWell tris-glycine precast gel (Thermo Fisher Scientific), and then transferred to a polyvinylidene difluoride membrane (Millipore) using a Trans-Blot Turbo Transfer System (Bio-Rad). Primary antibodies used were Sigma-Aldrich F1804 (anti-Flag) at 1:1000, Abcam ab1791 (anti-H3) at 1:15,000, and anti–CSR-1 (*22*) at 1:200. Secondary antibodies used for Western blots developed in a Bio-Rad ChemiDoc Touch system and exposed using Pierce ECL (Thermo Fisher Scientific) were GE Healthcare NA931 (sheep anti-mouse) at 1:2000 and the Jackson Laboratory 111035045 (goat anti-rabbit) at 1:15,000 when used with anti-H3 and 1:10,000 when used with anti–CSR-1 antibodies. Western blots developed using a LI-COR Odyssey Fc were done according to the manufacturer’s instructions using Odyssey Blocking Buffer and IRDye secondary antibodies at 1:15,000 (LI-COR).

### Single generation and transgenerational fertility assays

Gravid worms were hypochlorite treated, and their progeny, the P0 generation, were shifted to the appropriate temperature (25°C was used if not specifically stated) for all assays. Either the P0 generation or their progeny, the F1 generation, where indicated, was singled at the L2 to L3 stage, and their total progeny were counted. For transgenerational fertility assays, in addition to singling at the L2 to L3 stage, ~20 F1 worms were transferred to a single additional propagation plate so that their progeny could be singled at the subsequent generation.

### Differential interference contrast imaging of worms

Differential interference contrast images of worms were acquired on the Zeiss Axio Zoom V16 Fluorescence Stereo Microscope.

### Him assays

Worms were synchronized via hypochlorite treatment, and their progeny were plated at 20°C. At the L4 stage, 10 hermaphrodites were transferred to a new plate. The sex of their progeny was then scored.

### Immunofluorescence of the *C. elegans* germ line

Gravid adult *C. elegans* were dissected in egg buffer [118 mM NaCl, 48 mM KCl, 2 mM EDTA, 0.5 mM EGTA, and 25 mM Hepes (pH 7.4)], containing 15 mM sodium azide and 0.1% Tween 20, and then fixed in 1% formaldehyde in egg buffer for 10 s followed by a 1-min methanol fixation at −20°C. Primary mouse anti-FLAG antibody (Sigma-Aldrich, F1804) was used at 1:100, and rabbit anti–CSR-1 (*22*) was used at 1:200 (in fig. S1D) or 1:50 (in fig. S1C) in normal goat serum and PBST. The secondary antibody was used at 1:300 (Invitrogen Alexa Fluor 555 goat anti-mouse and 488 goat anti-rabbit) in PBST. All washes and staining were performed in suspension. Germ lines were stained with 4′,6-diamidino-2-phenylindole (0.5 mg/ml) and then mounted with VECTASHIELD (Vectorlabs, H-1000). Images were acquired on a Zeiss LSM700 confocal microscope at ×63 magnification. Image processing was performed using the Zen SP5 software. Images shown in fig. S1C were captured at ×100 magnification by a Leica Thunder Imaging System equipped with a 100× oil immersion, 1.4 numerical aperture objective, and a Leica K8 camera, and image processing was performed using the LAS X software.

### Chromatin immunoprecipitation on purified germline nuclei

A synchronized population of worms was obtained by hypochlorite treatment of gravid adult worms followed by overnight nutation of the embryos in M9. Worms were grown in liquid culture at 20°C according to the protocol described in (*51*) and collected at 56 hours (young adult stage). *morc-1::3xflag; aid::csr-1; psun-1::TIR1* (*aid::csr-1*) worms were grown in the presence of 50 μM auxin beginning at the L1 stage. At the 56-hour time point, the worms were cleansed via sucrose floatation (*51*). They were then live-crosslinked in 2.6% formaldehyde for 30 min at room temperature with nutation. The crosslinker was quenched with a 5-min nutation in glycine at a final concentration of 125 mM. Worms were washed in water and flash frozen in liquid nitrogen as ~1-ml pellets. Germline nuclei were purified according to the study of Han *et al*. (*31*) with some modifications. Briefly, frozen worms were ground in an MM400 Mixer Mill homogenizer (Retsch) for two rounds of 15 s at a frequency of 30^−1^ s. Frozen worm powder from each pellet was resuspended in 10 ml of nuclear purification buffer [50 mM Hepes (pH 7.5), 40 mM NaCl, 90 mM KCl, 2 mM EDTA, 0.5 mM EGTA, 0.2 mM dithiothreitol (DTT), 0.5 mM phenylmethylsulfonyl fluoride, 0.5 mM spermidine, 0.25 mM spermine, and 1 cOmplete ULTRA tablet (Roche) per 25-ml buffer] and allowed to chill on ice for 5 min. Resuspension was aided by 30 s of vortex at max speed. One additional round of 5 min on ice followed by 30-s max speed vortexing was performed. Samples were then spun at 30*g* for 5 min at 4°C. The supernatant was successively passed through two 40-μm filters (pluriSelect), then two 30-μm filters (pluriSelect), and lastly two 20-μm filters (pluriSelect). Nuclei were pelleted at 2400*g* for 6 min at 4°C, and the supernatant was removed. Purified germline nuclei were resuspended in 1-ml nuclear purification buffer, transferred to LoBind tubes (Eppendorf), repelleted at 1,500*g* for 5 min at 4°C, and flash frozen after removing the supernatant. Nuclei were resuspended in 1× radioimmunoprecipitation assay buffer (1× phosphate-buffered saline, 1% NP-40, 0.5% sodium deoxycholate, and 0.1% SDS) and nutated for 10 min at 4°C. Chromatin was sheared to a length of 100 to 500 bp using a Bioruptor Pico water bath sonicator (Diagenode) for three 3-min cycles, 30-s on/off. Crosslinked chromatin was immunoprecipitated overnight at 4°C with 2 μg of Flag antibody (Sigma-Aldrich, F1804) and then for 2 hours with 50 μl of Protein G Dynabeads (Invitrogen). Immunoprecipitated material was washed three times in LiCl buffer [100 mM tris-Cl (pH 7.5), 500 mM LiCl, 1% NP-40, and 1% sodium deoxycholate]. The crosslinking was then reversed in worm lysis buffer [0.1 M tris-Cl (pH 7.5), 0.1 M NaCl, 50 mM EDTA, and 1% SDS] with 6.8 μM proteinase K for at least 4 hours at 65°C in a Thermomixer (Eppendorf). DNA was extracted by phenol-chloroform and dissolved in TE buffer. Ribonuclease A (Invitrogen) treatment was performed for at least 2 hours at 37°C. For each immunoprecipitated sample, an input library was generated from 10% of the chromatin pre-IP and was de-crosslinked and extracted in parallel to the immunoprecipitated samples. Precipitated DNA was then used for library preparation using the Ovation Ultra Low System V2 kit (NuGEN) according to the manufacturer’s instructions and then sequenced on a NovaSeq 6000 Sequencer (Illumina).

### Chromatin immunoprecipitation on whole worms

Worms previously maintained at 20°C were hypochlorite treated and shifted to 25°C; these worms became the “P0” population. Worms were propagated at 25°C for four successive generations (F1 to F4); each generation was synchronized by hypochlorite treatment. When *csr-1* RNAi was used, it was fed to the worms beginning at the L1 stage only for the single generation before worm sample collection. Chromatin sonication, immunoprecipitation, de-crosslinking, and DNA extraction were performed as previously described (*21*). Antibodies used were Abcam 8898 (H3K9me3) and WAKO 300-95289 (H3K36me3). Precipitated DNA was then used for library preparation using the Ovation Ultra Low System V2 kit (NuGEN) according to the manufacturer’s instructions and then sequenced on a NovaSeq 6000 Sequencer (Illumina).

### RNA immunoprecipitation

The following strains were used for RIP: N2, JMC101 (*gfp::3xflag::csr-1*), and QK226 (JMC101 strain with the G560R mutation incorporated using CRISPR-Cas9). A synchronized population of worms was obtained by hypochlorite treatment of gravid adult worms followed by overnight nutation of the embryos in M9. Worms were grown in liquid culture at 20°C according to the protocol described in (*51*). Worms were collected at 56 hours (young adult stage) in water and flash frozen in liquid nitrogen as ~500-μl pellets. Frozen worms were homogenized in an MM400 Mixer Mill homogenizer (Retsch) for two rounds of 1 min at a frequency of 30^−1^ s. An estimated equal amount of worm powder per genotype was added to a 15-ml conical, and 1.5-ml IP buffer [10% glycerol, 10 mM EDTA, 30 mM Hepes (pH 7.4), 100 mM potassium acetate, 2 mM DTT, and 0.1% NP-40] was added. Worm powder was resuspended in the buffer by pipetting and vortexing. Worm lysis was transferred to a 2-ml LoBind microcentrifuge tube (Eppendorf) and spun at 18,000*g* for 5 min at 4°C. The lipid layer was aspirated off. Fifty microliters of the supernatant was reserved as the “input” sample and stored in 1-ml TRI reagent (Thermo Fisher Scientific) at −80°C. The rest of the worm lysis was mixed with 50-μl Dynabeads conjugated with anti-FLAG antibody (Sigma-Aldrich, F1804) and nutated at 4°C for 2 hours. Immunoprecipitated material was washed three times in cold IP buffer. Immunoprecipitated material (on Dynabeads) were stored in 1-ml TriReagent (Thermo Fisher Scientific) at −80°C. RNA extraction of the input and IP samples was performed as described below.

### RNA extraction

Worms were collected in TriReagent (Thermo Fisher Scientific) and subjected to three freeze-thaw cycles. Phase separation was achieved via 1-bromo-3-chloropropane, and the aqueous phase was precipitated in isopropanol at −80°C for 2 hours. To pellet RNA, samples were centrifuged at 21,000*g* for 30 min at 4°C. The pellet was washed three times in 75% ethanol and then resuspended in water.

### Quantitative reverse transcription PCR

cDNA for quantitation of mRNA levels was made from 500 ng of total RNA using Multiscribe Reverse Transcriptase (Applied Biosystems) and random hexamer primers in an Eppendorf Mastercycler Pro S6325 (Eppendorf). qPCR for mRNA levels was performed with Absolute Blue SYBR Green (Thermo Fisher Scientific) and normalized to *eft-2* or *him-3* [in experiments using the MORC-1 overexpression strain (*morcOE*)] using a CFX63 Real Time System Thermocycler (Bio-Rad). Specific primers used to measure mRNA levels are as follows: *morc-1:* GAAGCTGTGTCAAATGTGCCG and GAGAGTCGGACGATGATGGTG; codon-optimized *morc-1* transgene in *morcOE*: GAAGCTTGAGAAGGCCTCTGT and CGAGCCATTCCAAGACCATCA; *eft-2*: ACGCTCGTGATGAGTTCAAG and ATTTGGTCCAGTTCCGTCTG; *him-3*: CGACGGATTGAG-AGATGCGA and CGTTCGTGTCGATTCCGTTAT. Experiments were repeated in three biological replicates, although the figures show one biological replicate (with two technical replicates) unless otherwise stated.

### MORC-1 overexpression experimental scheme

*MorcOE* worms were maintained continuously on hygromycin (4 mg/ml) to select for worms inheriting the extrachromosomal array and neomycin (25 mg/ml; G418, Goldbio). To initiate an assay, *morcOE* worms were hypochlorite treated, and their progeny, the P0 generation, were seeded on NGM plates without antibiotic. P0 worms that lost the extrachromosomal array, by visual inspection for the absence of muscle-expressed mCherry (from *Pmlc-1::mCherry*), were singled. Loss of the extrachromosomal array was confirmed by genotyping (using primers aattttccagTCCAAGGCCG and GTC-TGGGTTCCCTCGTATGG). F1 progeny were then singled onto NGM plates that either contained neomycin (25 mg/ml) or no antibiotic. In addition to the singled worms, a plate of >30 worms of each condition (+/− neomycin) were plated for RNA extraction. The phenotype of the singled hermaphrodite was scored for uncoordinated (Unc) movement or wild-type movement and for neuronal mCherry-ON or mCherry-OFF by visual inspection under a fluorescence dissecting microscope (Leica). Fertility of each singled worm was measured. When the progeny of the worms plated for RNA extraction reached the adult stage, they were collected and stored in TriReagent (Thermo Fisher Scientific). This process was repeated at the F2 generation. However, at the F2 generation, worms that had been reared without antibiotic were showing substantial loss of the neuronal mCherry (*Prab-3::mCherry*); therefore, for the RNA collection samples, the worms were first manually separated according to their expression state of the neuronal mCherry (ON versus OFF).

### RNA-seq library preparation and sequencing

RNA-seq libraries were generated using the KAPA stranded mRNA-seq kit (Roche) according to the manufacturer’s instructions. Small RNA-seq libraries were made using the NEBNext Multiplex Small RNA Library Prep Set [New England Biolabs (NEB)] according to the manufacturer’s instructions. All RNA-seq libraries were sequenced on a NovaSeq 6000 Sequencer (Illumina).

### ATAC-seq

Transposase-accessible chromatin using sequencing (ATAC-seq) was performed on adult wild-type (N2) worms grown at 25°C. ATAC-seq was performed as previously described (*52*) and sequenced on a NovaSeq 6000 Sequencer (Illumina).

### Sequencing data analysis

For published datasets reanalyzed for this study (table S2), raw data were redownloaded from the Gene Expression Omnibus (GEO) database and processed using the same pipeline described here. All sequencing data were first checked for quality using fastqc v0.11.8 (*53*). Reads were filtered and trimmed to remove poor quality sequences and adapter sequences using Trim Galore v0.6.7 (*54*) with options --stringency 3 -q 25 --length 20. Reads were aligned to ce10/WBcel215 with the WS230 annotations, obtained from WormBase (https://wormbase.org). RNA-seq reads were aligned using STAR v2.7.9a (*55*) with options --outFilterMismatchNoverReadLmax 0.05 --alignIntronMin 70 --alignIntronMax 5000 --alignMatesGapMax 100000 --outFilterIntronMotifs RemoveNoncanonical --alignEnds-Type EndToEnd, while ChIP-seq and ATAC-seq reads were aligned using bowtie2 v2.3.4.3 (*56*) with options -N 0 -L 22 (-X 500 for paired-end data). PCR duplicates were removed using MarkDuplicates from the Picard tools suite (*57*). Alignment statistics for all libraries generated for this study are available in data S1.

### ATAC-seq data analysis

Aligned reads were analyzed using Genrich v.0.5 (*58*) to identify cut sites, excluding the mitochondrial chromosome. Cut site pileups from Genrich were used to make metaplots over gene promoters using DeepTools (*59*) computeMatrix and plotHeatmap functions.

### ChIP-seq data analysis

Fragment size was estimated using the run_spp function from the phantompeakqualtools (*60*, *61*) suite. Fragment size estimates averaged to ~175 bp across all samples, and this estimate was used for all analyses. Read coverage tracks normalized by counts per million (CPM) for each sample were generated using the bamCoverage function from DeepTools (*59*) suite v.3.5.1, using options --extendReads 175 --binSize 10 and --normalizeUsing CPM, and excluding the mitochondrial chromosome. In addition, coverage tracks of log_2_(IP/input) were also generated using the DeepTools bamCompare function again with --extendReads 175 --binSize 10 and --normalizeUsing CPM. For conditions with multiple replicates, average log_2_(IP/input) signal tracks were generated by (i) summing all IP tracks together using the DeepTools bigWigMerge function, (ii) summing all input tracks together (each IP track has a single matched input track), and (iii) calculating log_2_(sum IP/sum input) using DeepTools bigwigCompare. Average signal across specific genomic features (e.g., over promoters or 1-kb bins tiled genome-wide) were obtained using the DeepTools multiBigwigSummary function. The TSS region for a gene was defined as 1000 bp upstream to 400 bp downstream.

Signal peaks were identified using the MACS2 (*62*) v.2.2.7.1 callpeak function, with parameters -g 93260000 --broad -f BAM --nomodel --extsize 175. Peaks were called between each replicate IP and its matched input. For conditions with multiple replicates, consensus peaks were identified by first merging peaks across all replicates using bedtools (*63*) merge (“union peaks”). The union set of peaks was then filtered to only keep those that overlap at least 50% with each individual replicate: bedtools intersect -wa -a union_peaks.bed -b rep1_peaks.bed -f 0.5 -F 0.5 -e, producing the final set of consensus peaks. To calculate peak overlap with various genomic features (PCGs, promoters, repeats, TEs, etc.), BED files containing these different regions were first combined into a single file, and an extra column was added containing an integer ranking, such that TEs > PCG exons > PCG introns > non-PCG bodies > repeats > PCG promoter (2 kb up) > non-PCG promoter (2 kb upstream). Intervals in the consensus peak files were intersected with the regions BED file using bedtools intersect with options -a consensus_peaks.bed -b regions.bed -wao -f 0.1. Peaks overlapping multiple regions in the BED file were ranked by the ranking column, so that if, for example, both a TE and a PCG exon overlapped, the peak was assigned to “TE.” All peaks not overlapping any regions were assigned to category “other.”

Metaplots and heatmaps of ChIP-seq signal over genomic features/intervals were obtained using the DeepTools computeMatrix function, followed by plotProfile and/or plotHeatmap. *K*-means clustering of heatmap rows was performed using the --kmeans option in plotHeatmap, with optimal *k* chosen by visual inspection. To bin genes based on MORC-1 signal at the TSS in wild type, the difference in average log_2_(IP/input) signal between wild-type MORC-1 and the no-FLAG control over the TSS was calculated. The bins “none,” “weak,” “medium,” and “strong” were assigned to genes with signal difference ≤ 0, (0,0.5], (0.5,1], and > 1 respectively.

### RNA-seq data analysis

RNA-seq coverage tracks were generated using DeepTools (*59*) bamCoverage with options --binSize 10 --normalizeUsing CPM. Counts over genes were obtained using the htseq-count function from the HTSeq Python package (*64*) v2.0.2 with options --nonunique none -m intersection-strict and using the WS230 *C. elegans* annotations. Raw counts from each sample were combined into a counts matrix, and DESeq2 (*65*) was used to estimate expression changes between conditions [log_2_(fold change)] and to identify significantly differentially expressed genes. For *morcOE* samples, each condition was compared to the nontransgenic control. Metaplots and heatmaps of ChIP-seq signal over genomic features/intervals were obtained using the DeepTools computeMatrix function, followed by plotProfile and/or plotHeatmap. Transcripts per million (TPM) estimates were obtained using StringTie v.2.1.6 (*66*).

### sRNA-seq data analysis

Reads were first trimmed to remove adapter sequences using Trim Galore (*54*) with options --illumina --max_length 35 --length 18 -q 0 --stringency 3, retaining only reads between 18 and 35 bp posttrimming. Reads were further split according to size: 22 nt (CSR-1 and WAGO siRNAs plus microRNAs), 21 nt (PRG-1 piRNAs), and 26 nt (ERGO-1 and ALG-3/4-dependent siRNAs), and aligned to ce10 using bowtie2 (*56*) in --end-to-end --very-sensitive mode. Uniquely mapped reads were extracted (mapQ ≥ 2) and converted into strand-specific coverage tracks using DeepTools (*59*) bamCoverage --binSize 10 --normalizeUsing CPM --filterRNAstrand (forward/reverse). Counts over genes were obtained using the htseq-count function from the HTSeq Python package (*64*) v2.0.2 with options --nonunique none -m intersection-strict and using the WS230 *C. elegans* annotations, and log_2_(fold change) estimates between wild type and mutants were estimated using DESeq2 (*65*).

### RIP-seq analysis

Raw sequencing data was initially processed as for small RNA sequencing (sRNA-seq) analysis to obtain counts of different sRNA size classes over genes and coverage tracks. Where relevant, library size for normalization or comparison purposes was determined to be the number of uniquely aligned reads between 18 and 26 bp long. DESeq2 was used to compare read counts of IP versus input samples and obtain log2 fold change estimates of IP/input. Metaplots of read coverage were made using DeepTools, averaging the tracks for all three replicates.

### GO analysis

GO analysis for enriched GO terms was performed using DAVID (fig. S20B) (*67*). Tissue enrichment analysis (figs. S10A and S18B) was performed using the WormBase Enrichment Analysis tool (*68*).

### Genome browser images

Images of signal tracks were taken using the Integrative Genomics Viewer (*69*).

### Published datasets

Additional published datasets used in this study are shown in table S2. Some of these were redownloaded and reanalyzed using the same analysis pipeline described above, and mapping statistics for these libraries are available in data S1. Processed data are available in data S2.

### Data plotting

Heatmaps and metaplots were made using DeepTools (*59*). Other plots were made using Stata version 14 (*70*).

## Supplementary Materials

### The PDF file includes:

Supplementary Text Figs. S1 to S20 Tables S1 and S2 Legends for data S1 to S4 References
