# Supplementary material for: Regulation of MORC-1 is key to the CSR-1–mediated germline gene licensing mechanism in C. elegans
Source: Sci Adv. 2025 Jun 20;11(25):eado4170. doi: 10.1126/sciadv.ado4170 (PMC12180489; doi:10.1126/sciadv.ado4170)
Supplement: Supplementary file 1 — Supplementary Text Figs. S1 to S20 Tables S1 and S2 Legends for data S1 to S4 References [file sciadv.ado4170_sm.pdf]

Science Advances
AAAS

Supplementary Materials for

Regulation of MORC-1 is key to the CSR-1–mediated germline gene licensing mechanism in *C. elegans*

Jessica A. Kirshner *et al.*

Corresponding author: Steven E. Jacobsen, jacobsen@ucla.edu; John K. Kim, jnkim@jhu.edu

Sci. Adv. **11**, eado4170 (2025)
DOI: 10.1126/sciadv.ado4170

The PDF file includes:

Supplementary Text
Figs. S1 to S20
Tables S1 and S2
Legends for data S1 to S4
References

Other Supplementary Material for this manuscript includes the following:

Data S1 to S4

Supplementary Text

morc-1 does not rescue the *csr-1* Him phenotype

Partial loss of the CSR-1 pathway, which enables worms to produce a small number of progeny but still recapitulates many other *csr-1* defects, also leads to a high incidence of males (Him) phenotype, due to defects in chromosome segregation (*3*). In our RNA-seq data, both *csr-1(G560R)* and *aid::csr-1* showed strong upregulation of a shared set of genes enriched for sperm- and male-related gene ontology terms (fig. S20A–C). We speculated that this could be due to the Him phenotype, and examined the percentage of male progeny in both of our *csr-1* mutants. Indeed, we observed elevated rates of males in our *csr-1(G560R)*, while *aid::csr-1* could not be evaluated due to low fertility on auxin (fig. S20D), but nonetheless showed the elevated expression of male-related genes by RNA-seq (fig. S20A–C). This suggests that both of our *csr-1* mutants display the Him phenotype.

Unlike the *csr-1* chromatin defects, CSR-1 target gene downregulation, and fertility defects (Fig. 1C, Fig. 4A–B), the Him defect was not rescued in the *csr-1(G560R); morc-1(-)* double mutant (fig. S20D). Instead, the defect was mildly enhanced (fig. S20D). Consistent with this, the set of genes that are highly upregulated in *csr-1(G560R)*, which are enriched for male-related genes (fig. S20A–C), were further upregulated in the *csr-1(G560R); morc-1(-)* double mutant (fig. S20E). These genes are also mildly upregulated compared to wild-type in a published *morc-1* RNA-seq dataset (*21*), suggesting they may normally be repressed by MORC-1 (fig. S20E), which would explain why the *csr-1* Him phenotype is mildly enhanced in the *csr-1(G560R); morc-1(-)* double mutant. These data suggest that the chromosome segregation defects in *csr-1* are in a separate pathway than the defects rescued by *morc-1(-)*. This is consistent with previous work showing that ectopic upregulation of a different putative CSR-1 slicing target, *klp-7*, is the primary cause of chromosome segregation defects in *csr-1* (*14*).

Additional validation of *morcOE* lines

To validate our RNA-seq data from the *morcOE* lines, we checked the expression of both the integrated transgene and the extrachromosomal array, as well as endogenous *morc-1*, across all our RNA-seq samples (Fig. S17A–C) First, we confirmed that endogenous *morc-1* levels are similar across all of these lines as well as in the non-transgenic control, confirming that the *morcOE* worms have normal expression from the endogenous *morc-1* locus regardless of transgene expression (fig. S17B). We next confirmed that genes from the extrachromosomal array were expressed as expected. The extrachromosomal array contains both the piRNAi construct and the muscle mCherry, and should be lost after the P0 generation, as worms are manually selected for negative muscle mCherry expression (Fig. 5A, see methods). Expression of both genes was only detectable in the P0 generation, as expected (fig. S17C, see methods). Finally, the integrated transgene contains both a neuronal mCherry marker and the codon-optimized *morc-1*. We confirmed that both genes are expressed in all mCherry positive samples, but not in the mCherry negative samples, as expected. More generally, both genes show similar expression patterns, indicating that neuronal mCherry is a good proxy for transgene *morc-1* expression (fig. S17C).

All *morcOE* worms, regardless of the status of *morc-1* transgene expression, shared a set of largely overlapping upregulated genes (fig. S18A–C), indicating that these genes are not upregulated due to *morc-1* overexpression. These genes were not significantly enriched for germline genes or any other significant gene ontology terms (fig. S18B–C).

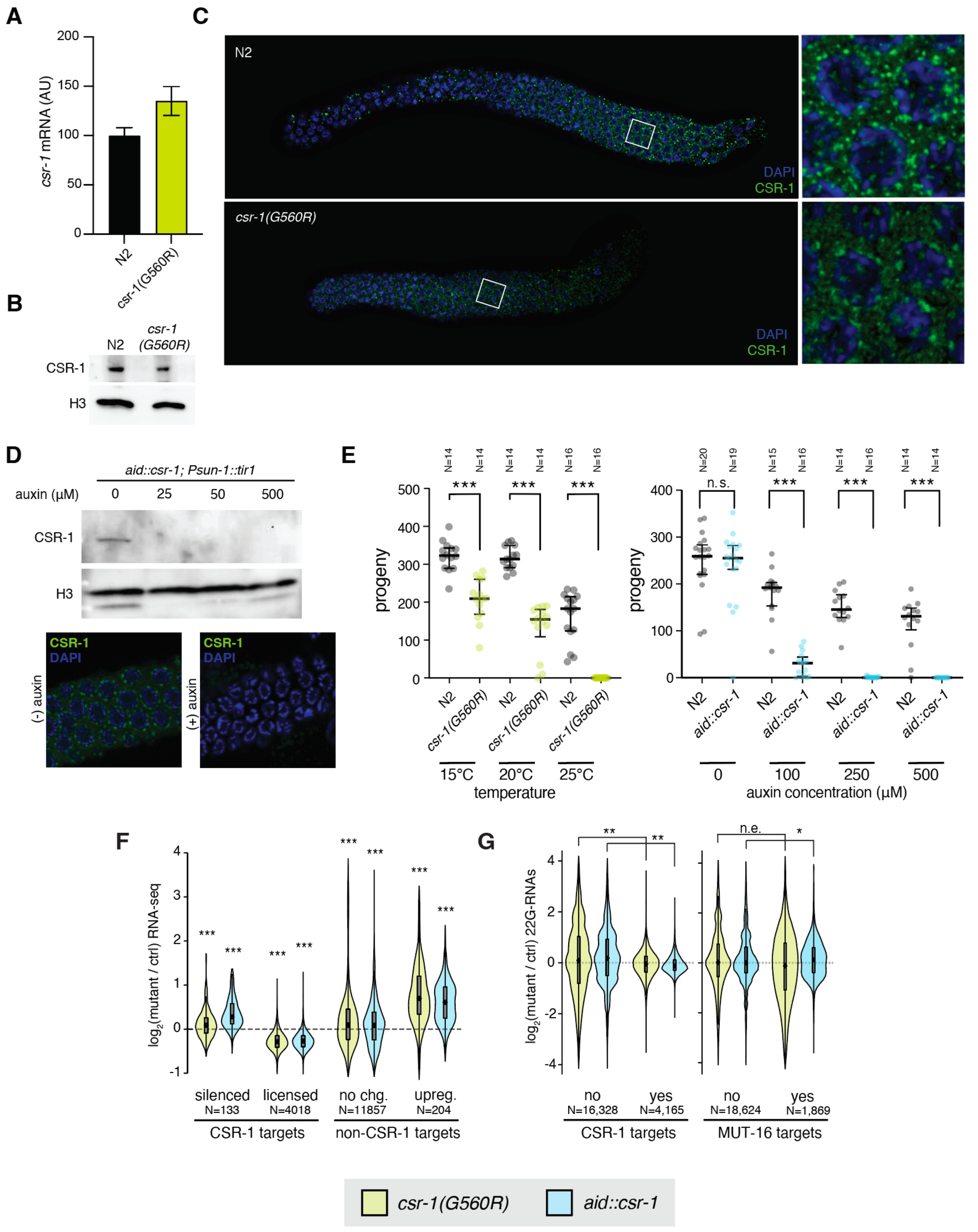

Fig. S1. Validation of *csr-1(G560R)* and *aid::csr-1*. **(A)** mRNA qPCR of *csr-1* expression in wild-type (N2) and *csr-1(G560R)*. Error bars represent standard error of the mean for three biological replicates, each with two technical replicates. **(B)** Western blot for CSR-1 (native

antibody) in wild-type and *csr-1(G560R)*. H3 shown as a loading control. **(C)** Immunofluorescence of CSR-1 (green) in germlines extruded from wild-type and *csr-1(G560R)* worms. (left) whole germline images, (right) magnified view of boxed region on the left. Worms are co-stained with DAPI (blue). (**D**) (top) Western blot for CSR-1 (native antibody) in *aid::csr-1* worms exposed to increasing concentrations of auxin. (bottom) Immunostaining of CSR-1 in dissected germlines from *aid::csr-1* worms on 0 μM [(-) auxin] or 100 μM auxin [(+) auxin]. (**E**) Number of progeny in wild-type or *csr-1(G560R)* at permissive vs. restrictive temperature (left), and *aid::csr-1* worms exposed to increasing concentrations of auxin vs. wild-type control (right). Each point represents a single worm (n ≥14 worms assayed per genotype/condition). n.s. = $p > 0.05$, *** = $p < 0.001$, one-tailed t-test. (**F**) Average RNA-seq expression $\log_2$ fold change vs. control of *csr-1(G560R)* (control = N2) and *aid::csr-1* [(+) auxin] (control = *aid::csr-1* [(-) auxin]) worms, over CSR-1 silenced targets vs. licensed targets, *csr-1(-)* upregulated non-targets (“upreg.”) vs. all other non-targets (“no chg.”). Silenced, licensed, unchanged and upregulated genes were identified in Gerson-Gurwitz *et al.* (*14*). Significance measured by two-tailed t-test, n.s. = not significant, *** = $p < 0.0001$, with null hypothesis that $\log_2$(fold change) = 0. (**G**) Average $\log_2$ fold change of *csr-1(G560R)* and *aid::csr-1* vs. control of 22G-RNAs over CSR-1 targets and MUT-16 targets vs. non-targets (*3*). Effect size measured using Cohen’s d. n.e. = no/minimal effect ($|d| < 0.2$), * = $|d| > 0.2$, ** = $|d| > 0.5$, *** = $|d| > 0.9$, **** = $|d| > 1.5$. Note that these comparisons give $p \sim 0$ by Student’s t-test due to large sample size.

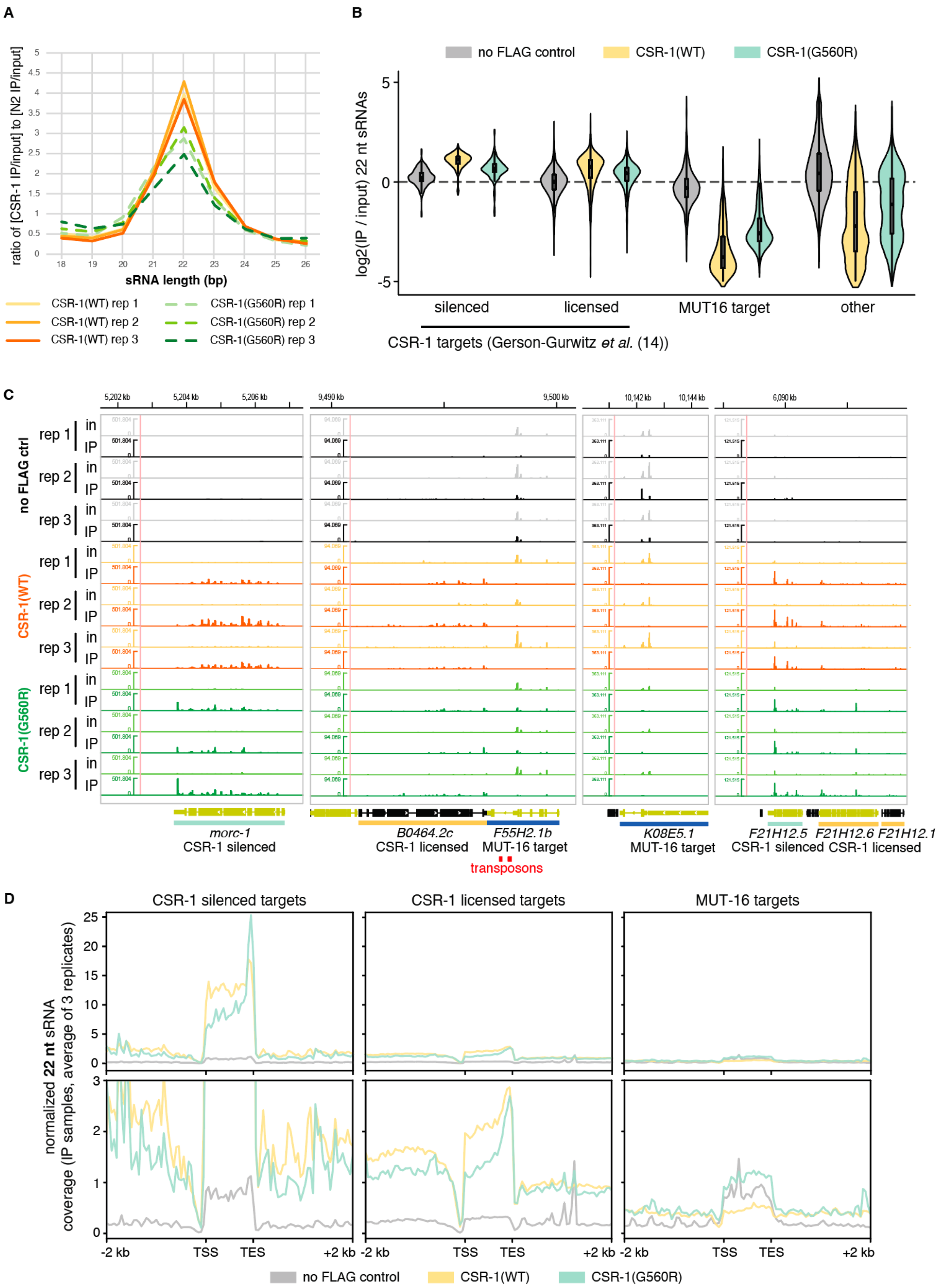
A
ratio of [CSR-1 IP/input] to [N2 IP/input]
sRNA length (bp)
CSR-1(WT) rep 1
CSR-1(WT) rep 2
CSR-1(WT) rep 3
CSR-1(G560R) rep 1
CSR-1(G560R) rep 2
CSR-1(G560R) rep 3
B
no FLAG control
CSR-1(WT)
CSR-1(G560R)
log2(IP / input) 22 nt sRNAs
silenced
licensed
MUT16 target
other
CSR-1 targets (Gerson-Gurwitz et al. (14))
C
no FLAG ctrl
CSR-1(WT)
CSR-1(G560R)
rep 1
rep 2
rep 3
in
IP
morc-1
CSR-1 silenced
B0464.2c
CSR-1 licensed
F55H2.1b
MUT-16 target
transposons
K08E5.1
MUT-16 target
F21H12.5
CSR-1 silenced
F21H12.6
CSR-1 licensed
F21H12.1
D
CSR-1 silenced targets
CSR-1 licensed targets
MUT-16 targets
normalized 22 nt sRNA coverage (IP samples, average of 3 replicates)
-2 kb
TSS
TES
+2 kb
no FLAG control
CSR-1(WT)
CSR-1(G560R)

Fig. S2: CSR-1-bound 22 nt small RNAs are less efficiently bound by CSR-1(G560R). (**A**) Enrichment of indicated length sRNAs in IPs of 3xFlag-tagged CSR-1(WT) and CSR-1(G560R), normalized first to matched input samples, then to matched no-Flag control (N2). Abundances were all normalized to the total number of sRNAs between 18–26 bp in each library, prior to calculating enrichments. (**B**) Fold change enrichment of 22 nt sRNAs in IP samples relative to input samples, for sRNAs overlapping either silenced or licensed CSR-1 targets, as previously defined by Gerson-Gurwitz *et al.* (*14*). Log_2 fold change estimates were calculated using DESeq2. (**C**) Example genome browser images of 22 nt sRNA coverage (normalized to library size, considering all sRNAs between 18–26 bp) over CSR-1 silenced or licensed targets, as well as MUT-16 targets. *morc-1* shown on the left. Matched input and IP samples shown side by side for 3 replicates per genotype. (**D**) Metaplots of average coverage (normalized to total library size, considering all sRNAs between 18–26bp) of 22 nt sRNAs in indicated IP samples, over CSR-1 silenced and licensed targets, as well as MUT-16 targets. Each line represents average of 3 experiments. Bottom plots are the same as the top, but with y-axis limited to 3.

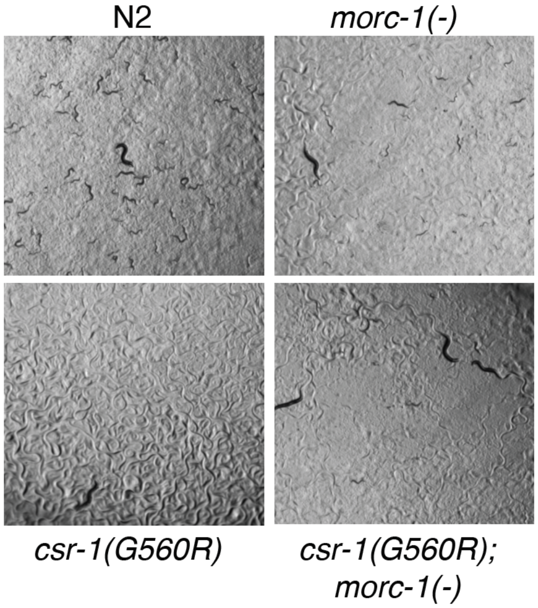

Fig. S3. Rescue of *csr-1(G560R)* fertility by *morc-1.* Example DIC images of the parents and progeny of 10 parental worms of each genotype grown at the *csr-1(G560R)* nonpermissive temperature of 25°C.

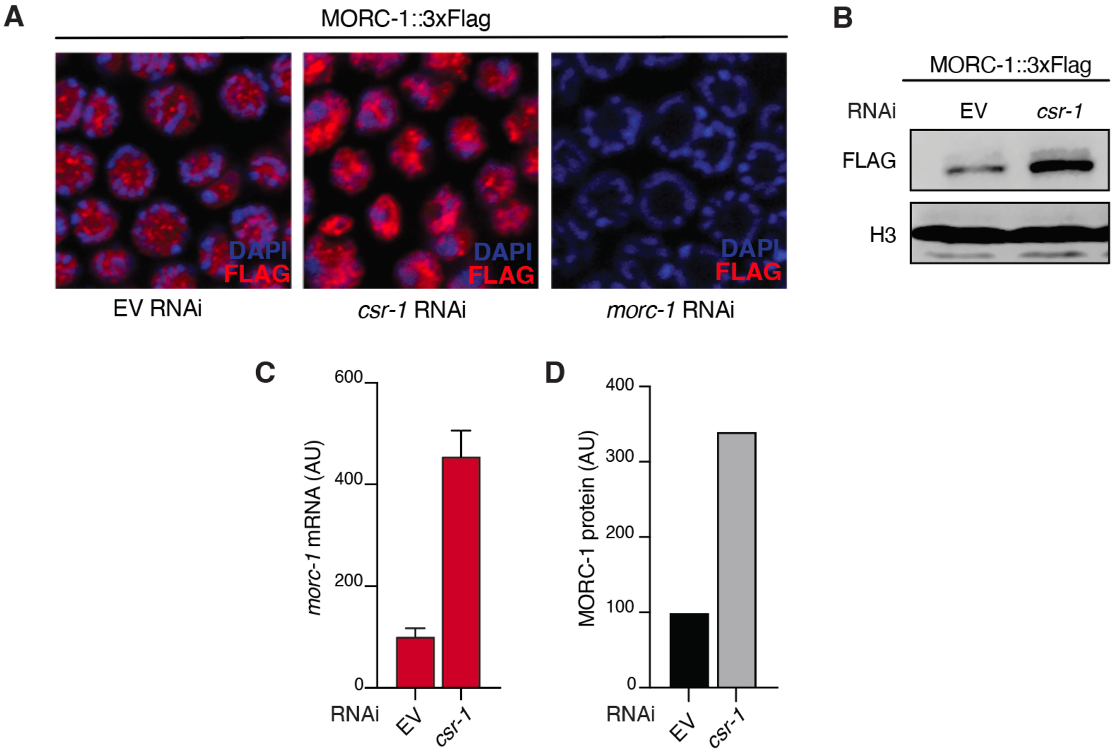

Fig. S4. Control of *morc-1* expression by *csr-1* RNAi. (**A**) Immunofluorescence of MORC-1::3xFlag treated with *csr-1* or *morc-1* RNAi compared to empty vector (EV) control, in dissected germlines. Flag signal shown in red, DAPI in blue. (**B**) Western blot of MORC-1::3xFlag worms treated with RNAi against *csr-1* or an empty vector control (EV). (**C**) *morc-1* mRNA quantification by qPCR for wild-type (N2) worms treated with RNAi against *csr-1* or an empty vector control (EV). Error bars represent standard deviation of two technical replicates. (**D**) Quantification of protein levels in the western blot shown in (B).

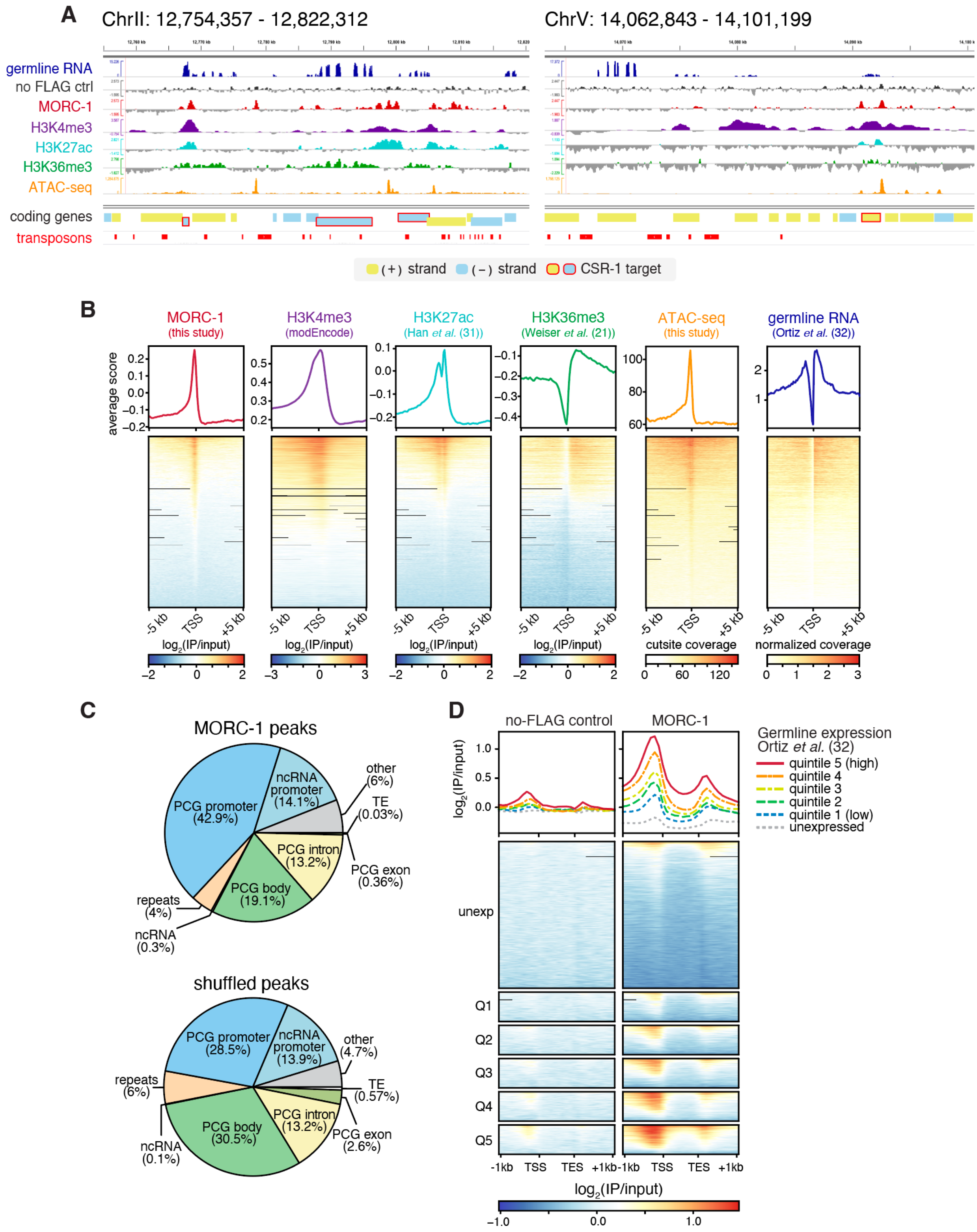

Fig. S5. MORC-1 localization in *C. elegans*. (**A**) Example genome browser images showing MORC-1::3xFlag localization alongside other published histone modification ChIP-seq datasets (see Table S2), germline expression from Ortiz *et al.* (*32*), and chromatin accessibility as evaluated

by ATAC-seq (this study). (**B**) Metaplots and heatmaps of MORC-1::3xFlag, published histone modification ChIP-seq (see Table S2), chromatin accessibility (this study), and germline RNA-seq (*32*) over gene transcriptional start sites (TSSs), ranked by MORC-1::3xFlag levels (leftmost heatmap). All heatmaps have same row order. (A-B) For ChIP-seq, the plotted values are log IP signal normalized to input (log_2(IP/input)), for ATAC-seq the plotted values are the number of ATAC-seq cut sites (see methods), and for RNA-seq the values shown are normalized coverage. (**C**) Distribution of MORC-1 peaks across different genome annotations. PCG = protein-coding gene. Promoters are defined as the region from 2 kb upstream of TSS to the TSS. Peaks in PCG bodies preferentially over introns or exons (> 70% of length of peak) are listed explicitly as 'PCG intron' or 'PCG exon', while all other peaks in PCG bodies are listed as 'PCG body.' True peak distribution was compared to the distribution of randomly shuffled peaks. (**D**) Heatmaps of ChIP-seq signal from no-Flag control (left) or MORC-1::3xFlag (right), over protein-coding genes binned into quintiles by germline expression level from Ortiz *et al.* (*32*). Genes detected in Ortiz *et al.* (*32*) as germline-expressed were divided into quintiles Q1-Q5 based on average TPM levels in the Ortiz *et al.* (*32*) RNA-seq, with Q5 containing the most highly expressed genes, while unexpressed genes ("unexp") were kept as a separate group.

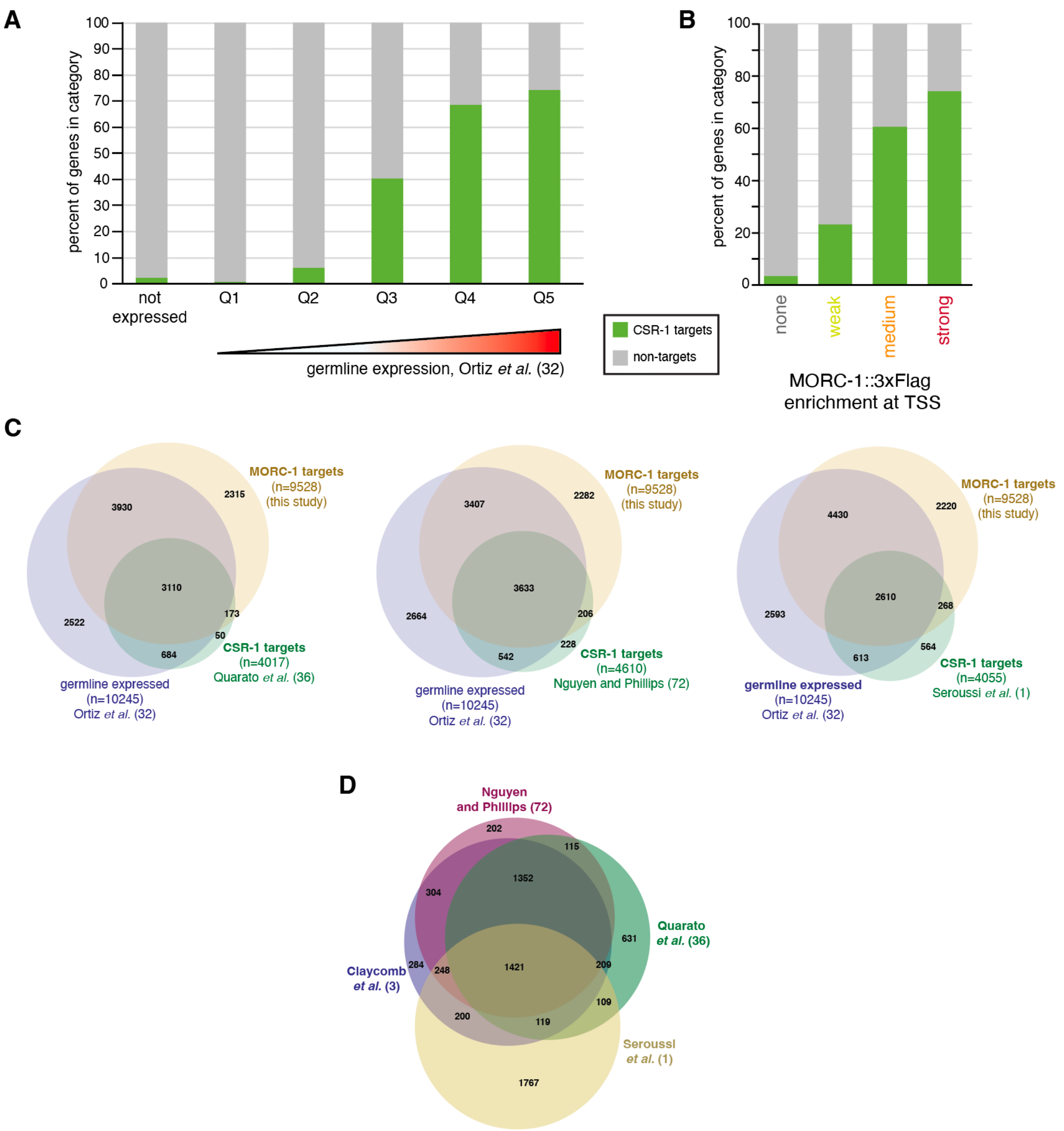

Fig. S6. CSR-1 targets are highly expressed in the germline and strongly bound by MORC-1. (**A**) Percent of genes binned by germline expression level that are CSR-1 targets identified in Claycomb *et al.* (*3*). Genes detected in Ortiz *et al.* (*32*) as germline-expressed were divided into quintiles based on average TPM levels, with Q5 containing the most highly expressed genes. Undetected/unexpressed genes were kept as a separate category. **(B)** Percent of genes binned by MORC-1 enrichment at the TSS (same bins as Fig. 2E) that are CSR-1 targets identified in Claycomb *et al*. (*3*) **(C)** Overlap between CSR-1 target genes (green) identified in three different studies (*1*, *36*, *72*), germline expressed genes (purple) from Ortiz *et al.* (*32*), and MORC-1 targets (yellow, this study) identified in this study based on MORC-1 enrichment over the promoter and TSS. *** = p ~ 0, hypergeometric test. **(D)** Overlap between lists of CSR-1 targets identified in four different studies (*1*, *3*, *36*, *72*).

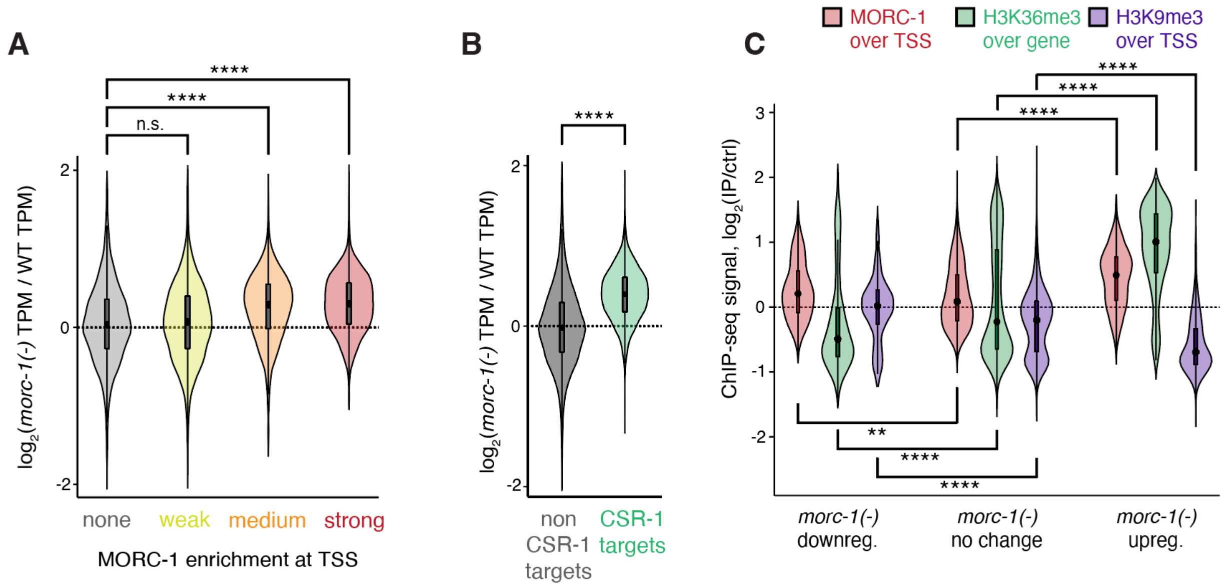

Fig. S7. Relationship between MORC-1 occupancy and *morc-1(-)* expression changes. (**A–B**) Expression change in *morc-1(-)* from Weiser *et al.* (*21*), as a function of MORC-1 levels at the transcription start site (TSS) in wild-type animals (A), or CSR-1 targets vs. non-targets (B). A small number of genes (N=68) with y-values outside of [-2,2] not shown. (**C**) Wild-type ChIP-seq signal ($\log_2$(IP/input)) of MORC-1::3xFlag (this study) over TSS, H3K36me3 (*21*) over gene body, and H3K9me3 (*21*) over TSS, comparing genes significantly upregulated (N=1,302), downregulated (N=403), or unchanged in *morc-1(-)* based on Weiser *et al.* (*21*) RNA-seq. All data from Weiser *et al.* (*21*) are from the F4 generation of *morc-1(-)* worms maintained at 25°C. (A-C) Significance by two-tailed t-test: n.s. = not significant, * = $p < 0.01$, ** = $p < 0.001$, *** = $p < 0.0001$, **** = $p < 0.00001$.

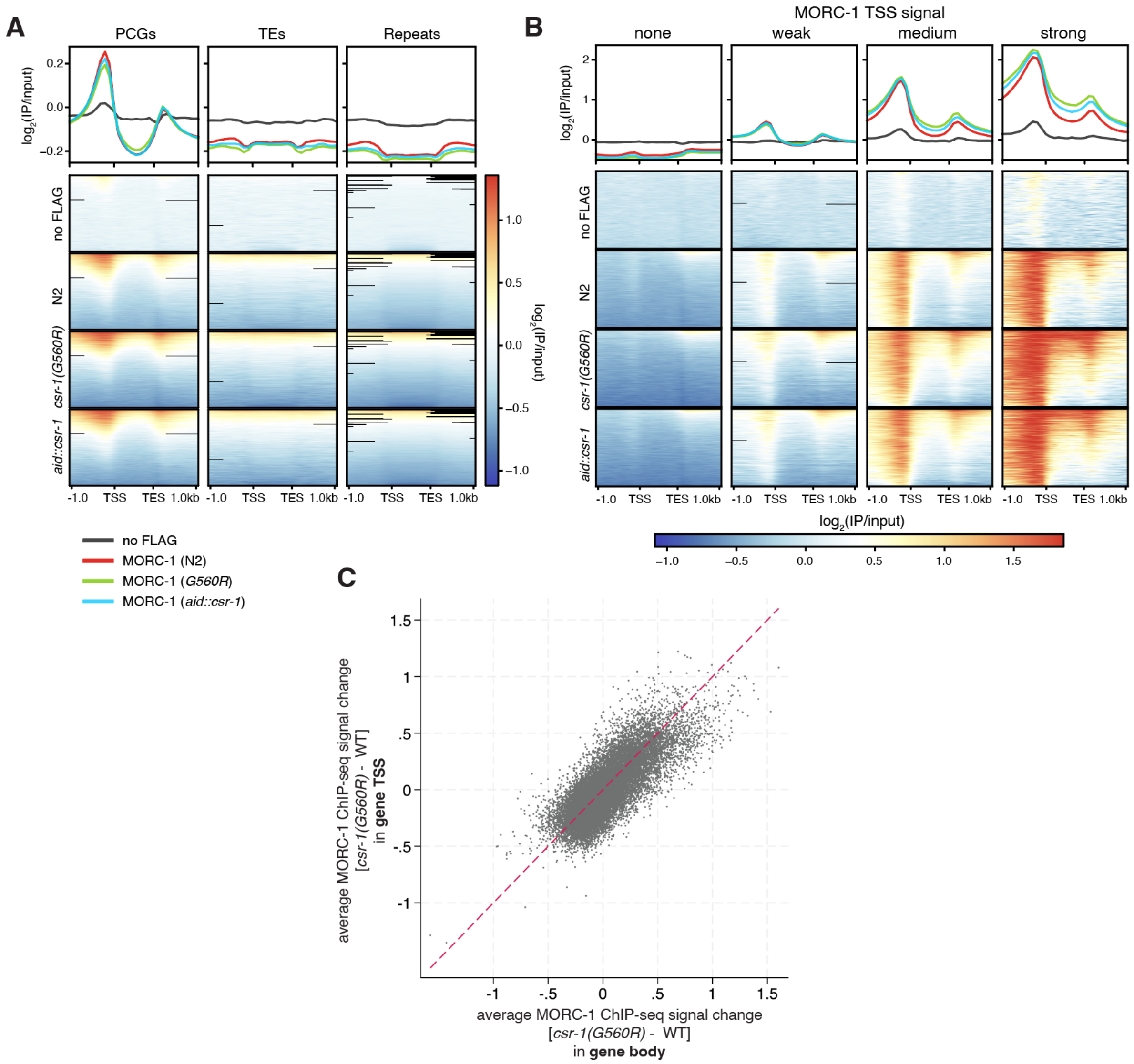

Fig. S8. MORC-1 further accumulates at targets when overexpressed. (**A**) Metaplots and heatmaps showing MORC-1::3xFlag localization ($\log_2$(IP/input)) in wild-type and both *csr-1(G560R)* and *aid::csr-1* [(+) auxin], over protein coding genes (PCGs), transposons (TEs), and repeat regions. No-Flag control sample (wild-type worms lacking the 3xFlag) shown for reference. Legend for metaplots shown at bottom left. (**B**) Metaplots and heatmaps showing MORC-1 localization in wild-type and both *csr-1(G560R)* and *aid::csr-1* [(+) auxin], over genes divided by wild-type MORC-1 occupancy at TSS. Legend for metaplots same as A. **(C)** Scatterplot of change in MORC-1 ChIP-seq signal in *csr-1(G560R)* compared to wild-type (WT) in gene body (x-axis) or TSS region (y-axis). Red dotted line shows y = x.

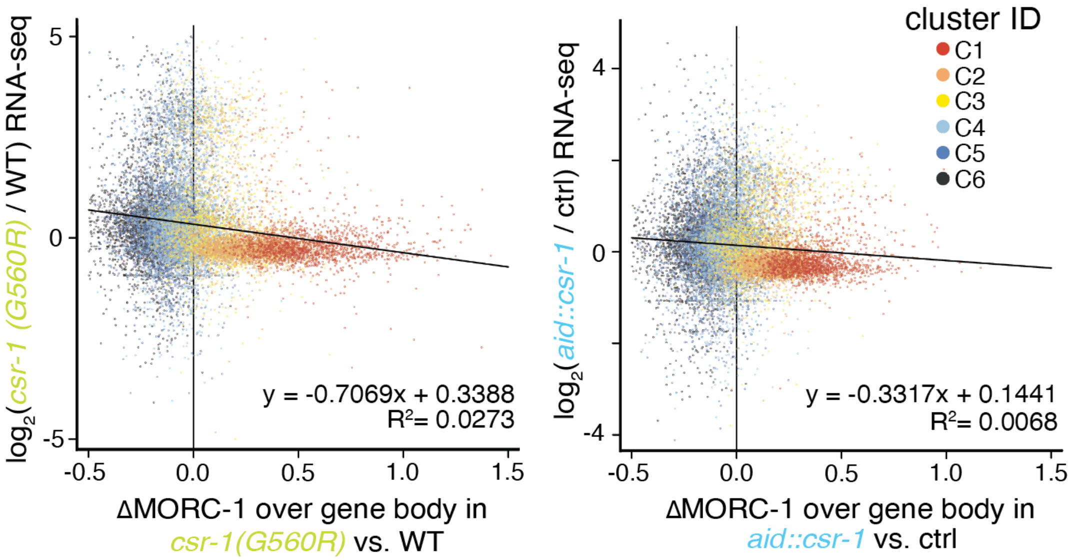

Fig. S9. Correlation between MORC-1 gain in *csr-1* and gene downregulation. Scatterplots of change in MORC-1 levels over the gene body in *csr-1(G560R)* (left panel) or *aid::csr-1* [(+) auxin] (right panel) compared to appropriate control, vs. the change in RNA-seq expression in the same mutant vs. control (DESeq2 estimated $\log_2$(fold change)). Genes are colored according to their cluster in Fig. 3B, with C1 (red) genes showing the highest MORC-1 gain in *csr-1*. Black line indicates linear regression best fit line; equation and R^2 shown at bottom right. For *csr-1(G560R)*, the control is N2 while for *aid::csr-1* all comparisons are between *aid::csr-1* worms treated with 1 mM auxin [(+) auxin] vs. 0 mM auxin [(-) auxin].

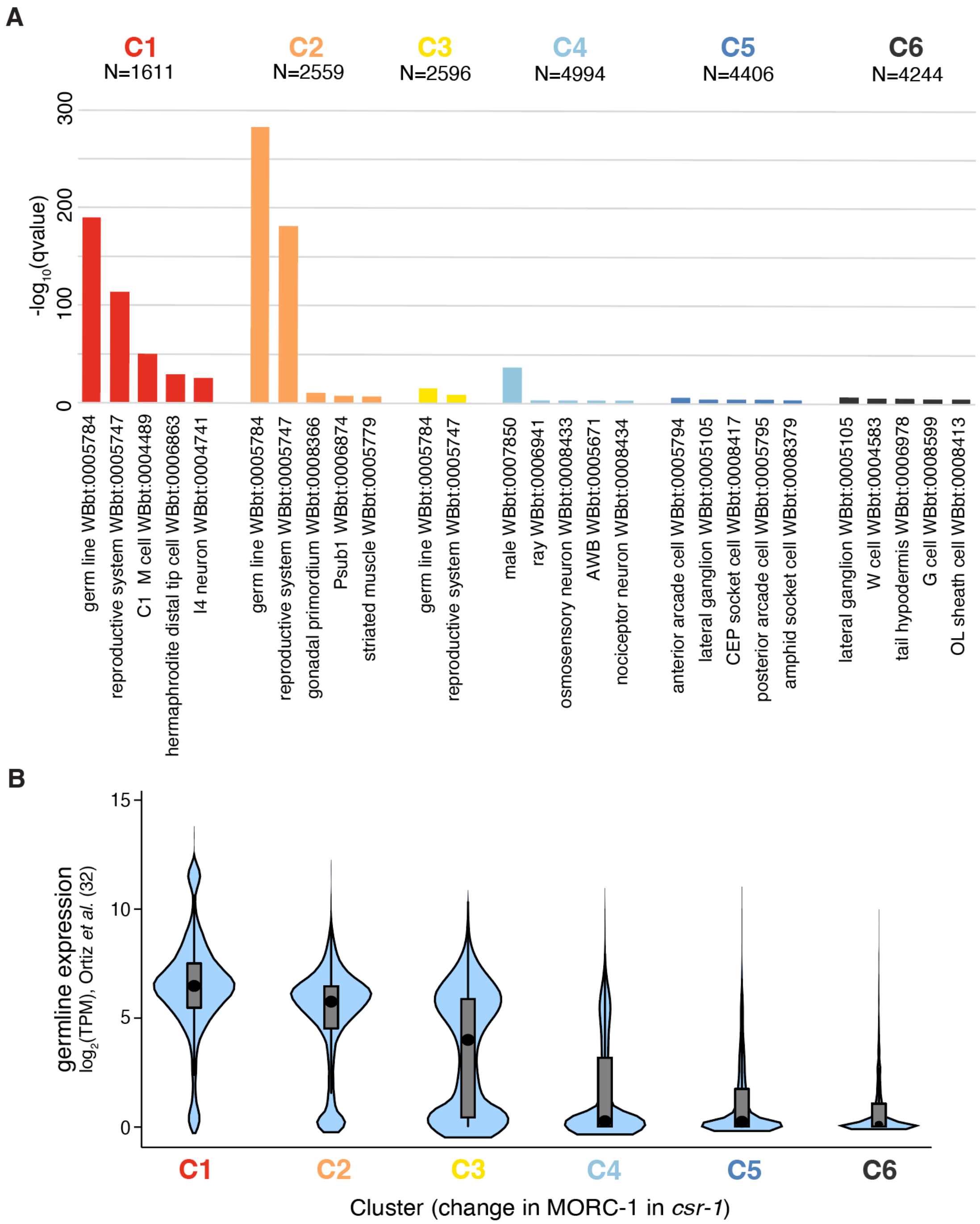

Fig. S10. Characterization of MORC-1 gain clusters. (A) Tissue enrichment analysis using the WormBase Enrichment Analysis tool (*68*) for genes in each cluster from Fig. 3B, with C1 (red) genes showing the highest MORC-1 gain in *csr-1*. **(B)** Distribution of average germline expression from Ortiz *et al.* (*32*) in each of the clusters from Fig. 3B.

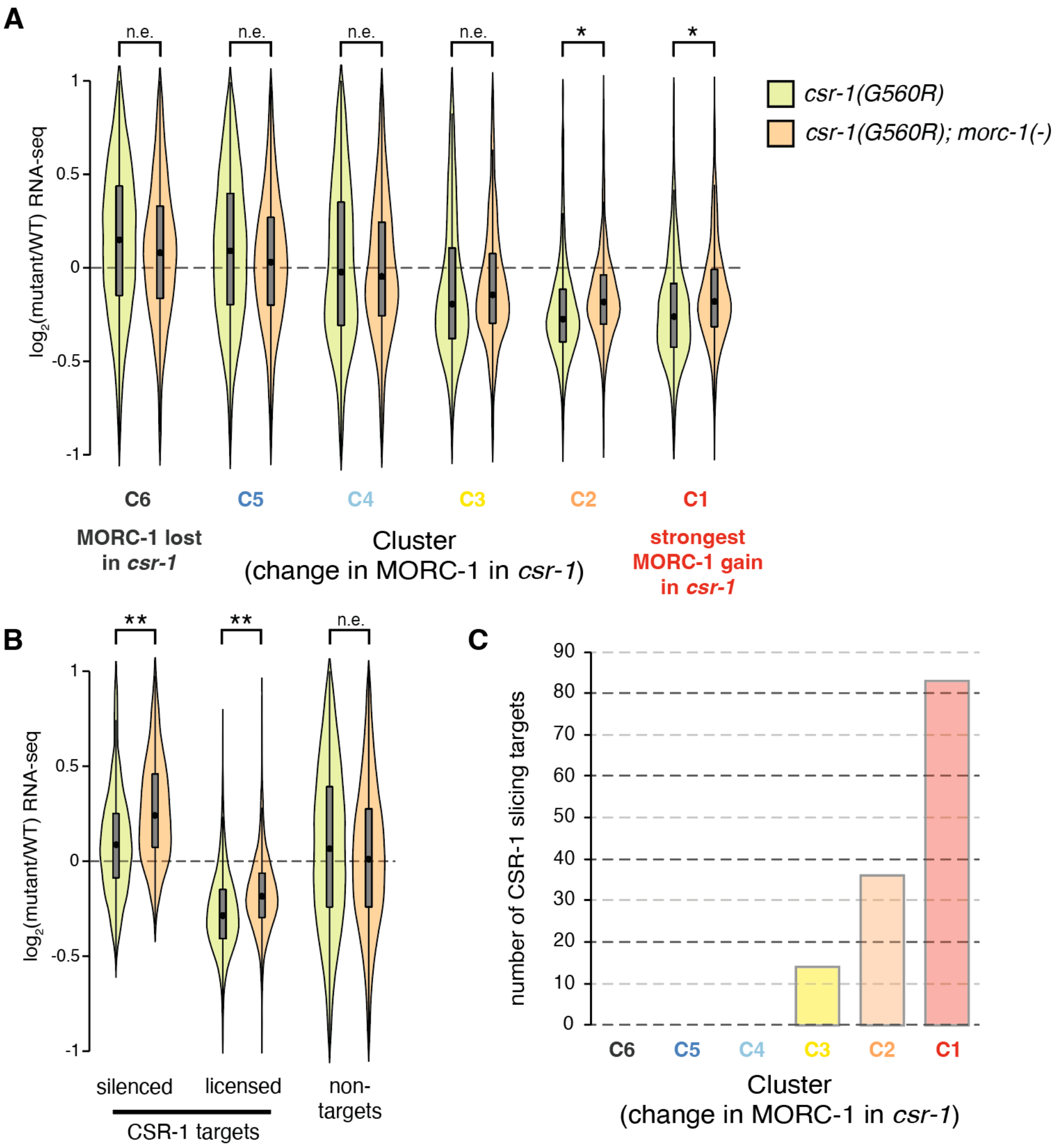

Fig. S11. Target gene expression defects in *csr-1* are rescued by *morc-1* at genes highly bound by MORC-1. (**A-B**) Distribution of $\log_2$ fold change expression values of indicated mutant over wild-type, estimated by DESeq2, over (A) genes clustered based on change in MORC-1 levels in both *csr-1* mutants vs. WT, using k-means clustering with k == 6 (Fig. 3B), and (B) CSR-1 targets vs. non-targets. Stars indicate effect size measured by Cohen's d: n.e. = no/minimal effect ($|d| < 0.1$), * = $|d| > 0.1$, ** = $|d| > 0.25$. (C) Number of CSR-1 silenced targets (N=133, Gerson-Gurwitz *et al.* (*14*)) that fall into each of the 6 *k*-means clusters based on change in MORC-1 levels in *csr-1* vs. WT (Fig. 3B).

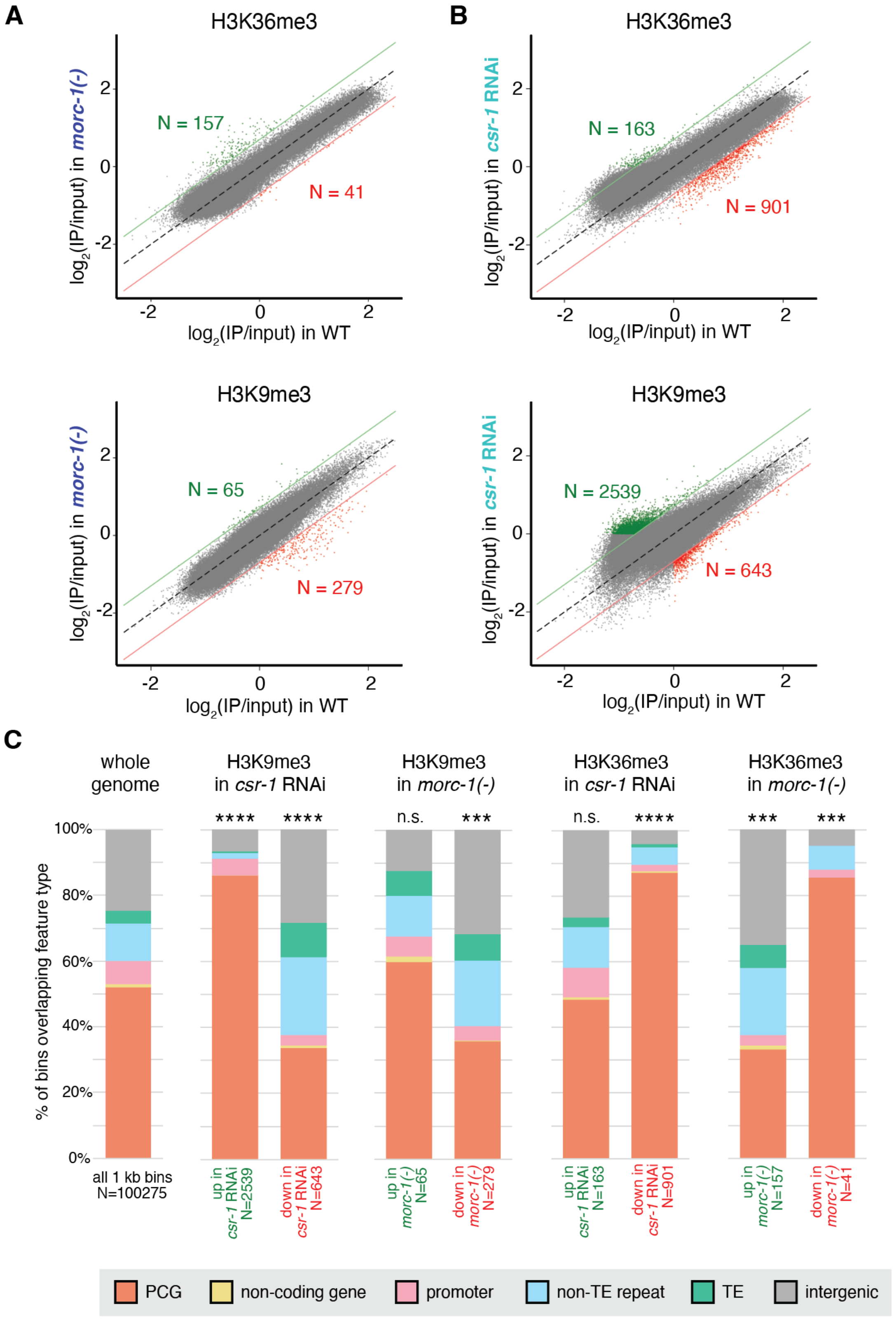

Fig. S12. Genome-wide chromatin changes in *morc-1(-)* and *csr-1* RNAi. (**A-B**) Scatterplots of average H3K36me3 and H3K9me3 ChIP-seq signal (log_2(IP/input)) in 1 kb bins tiled genome-wide. Wild-type (N2 + empty vector (EV) RNAi) signal is plotted against (A) *morc-1(-)* + EV

RNAi or (B) N2 + *csr-1* RNAi. Significantly different bins, identified as +/-0.7 difference in $\log_2$(IP/input) between wild-type and *morc-1(-)* or *csr-1* RNAi (truncated at 0), are highlighted in green and red (black dotted line: y = x line, green line: y = x + 0.7, red line: y = x – 0.7). (**C**) Distribution of genomic features overlapped by the 1 kb bins identified in A-B as having increased, decreased, or remained unchanged H3K9me3 or H3K36me3 in *csr-1* RNAi or *morc-1(-)*. Significant deviation from the genome-wide distribution (leftmost panel) was evaluated using a Chi-squared test. Significance testing: (A-B) **** = $p < 0.0001$, *** = $p < 0.001$, ** = $p < 0.01$, * = $p < 0.05$, n.s. = not significant.

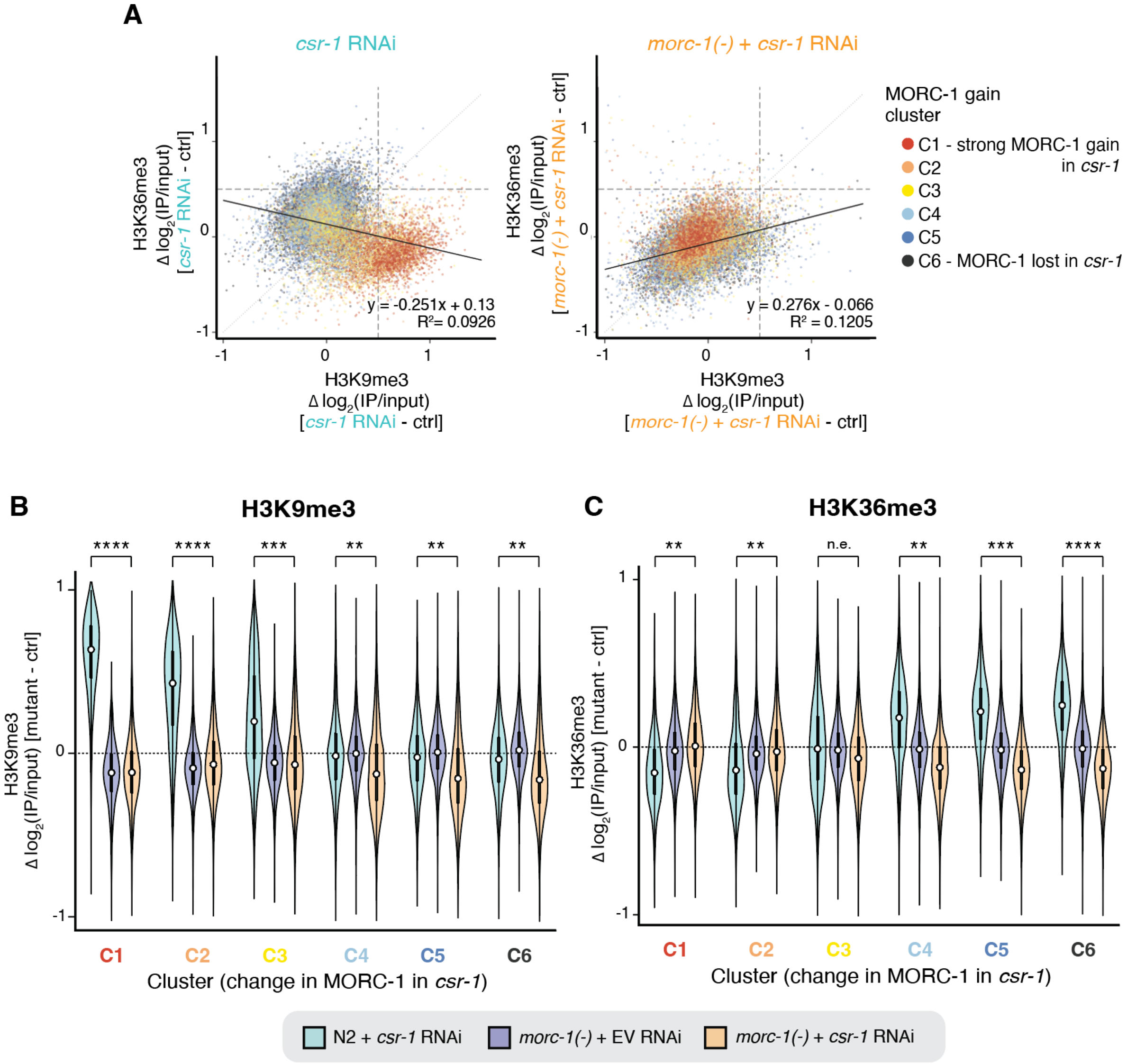

Fig. S13. Changes in H3K9me3 and H3K9me3 in *csr-1* RNAi correlate with each other and are rescued by *morc-1(-)*. (**A**) Scatterplots of change in H3K9me3 vs. change in H3K36me3 over gene bodies, in wild-type (N2) treated with *csr-1* RNAi or *morc-1(-)* treated with *csr-1* RNAi vs. control (N2 treated with empty vector (EV) RNAi). Points are colored based on that gene's *k*-means cluster from Fig. 3B, with C1 (red) genes showing the highest MORC-1 gain in *csr-1*. Black line shows linear regression line, with equation and R^2 at bottom right. (**B-C**) Distribution of change in (B) H3K9me3 and (C) H3K36me3 in indicated genotype + RNAi vs. control (in all cases, control is N2 + EV RNAi), over genes clustered based on change in MORC-1 levels in *csr-1* vs. WT, with C1 (red) genes showing the highest MORC-1 gain in *csr-1* (Fig. 3B). Cohen's d. n.e. = no/minimal effect ($|d| < 0.2$), * = $|d| > 0.2$, ** = $|d| > 0.5$, *** = $|d| > 0.9$, **** = $|d| > 1.5$.

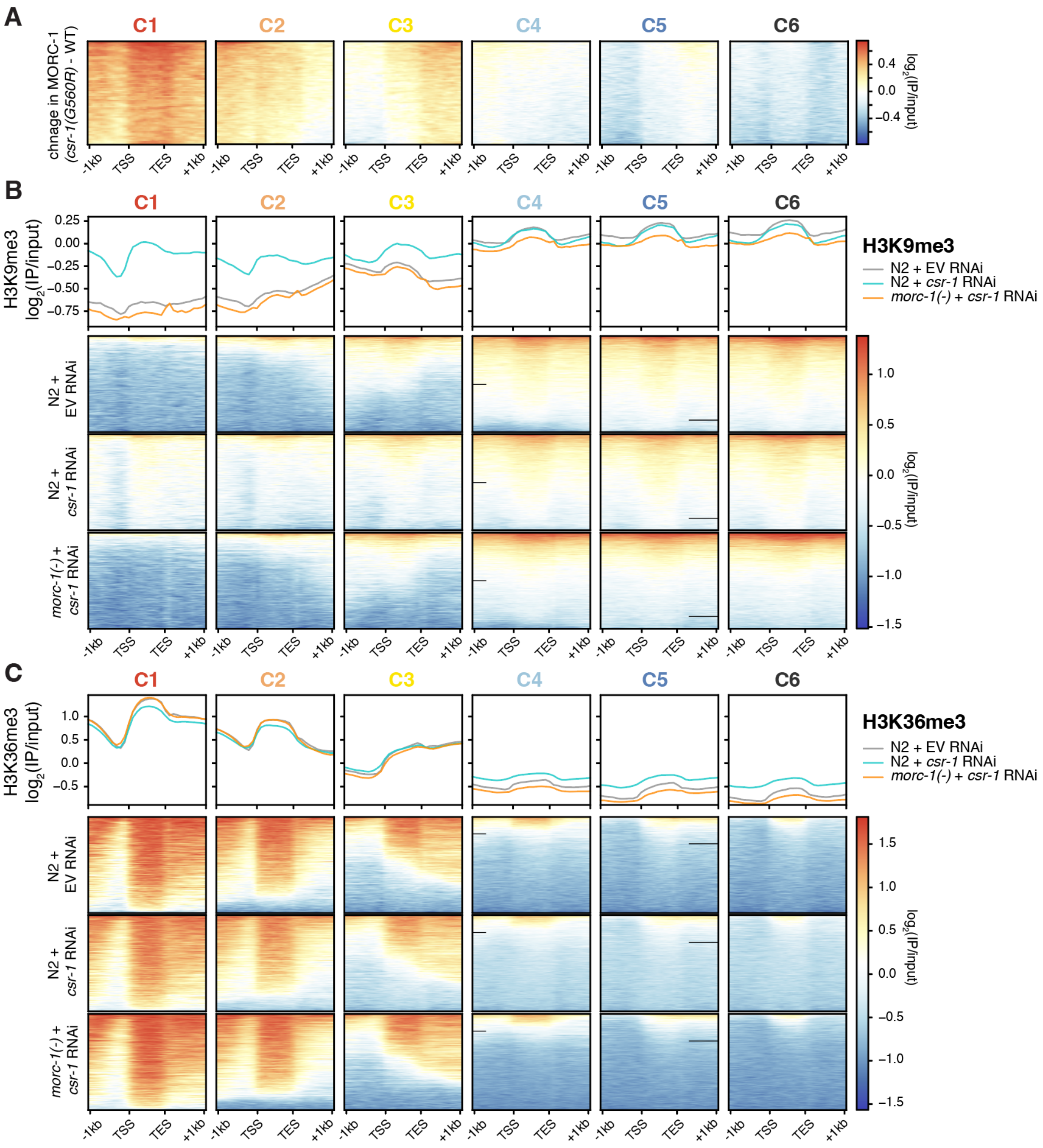

Fig. S14. Changes in chromatin environment as a function of MORC-1 gain in *csr-1(-)*. (**A**) Reproduction of heatmaps from Fig. 3B, showing pattern of MORC-1 gain in each of the 6 *k*-means clusters in *csr-1(G560R)*, with C1 (red) genes showing the highest MORC-1 gain in *csr-1*. (**B-C**) Metaplots and heatmaps of (B) H3K9me3 and (C) H3K36me3, over genes in the 6 *k*-means clusters from Fig. 3B, in wild-type worms treated with empty vector (EV) RNAi or *csr-1* RNAi, vs. *morc-1* worms treated with *csr-1* RNAi.

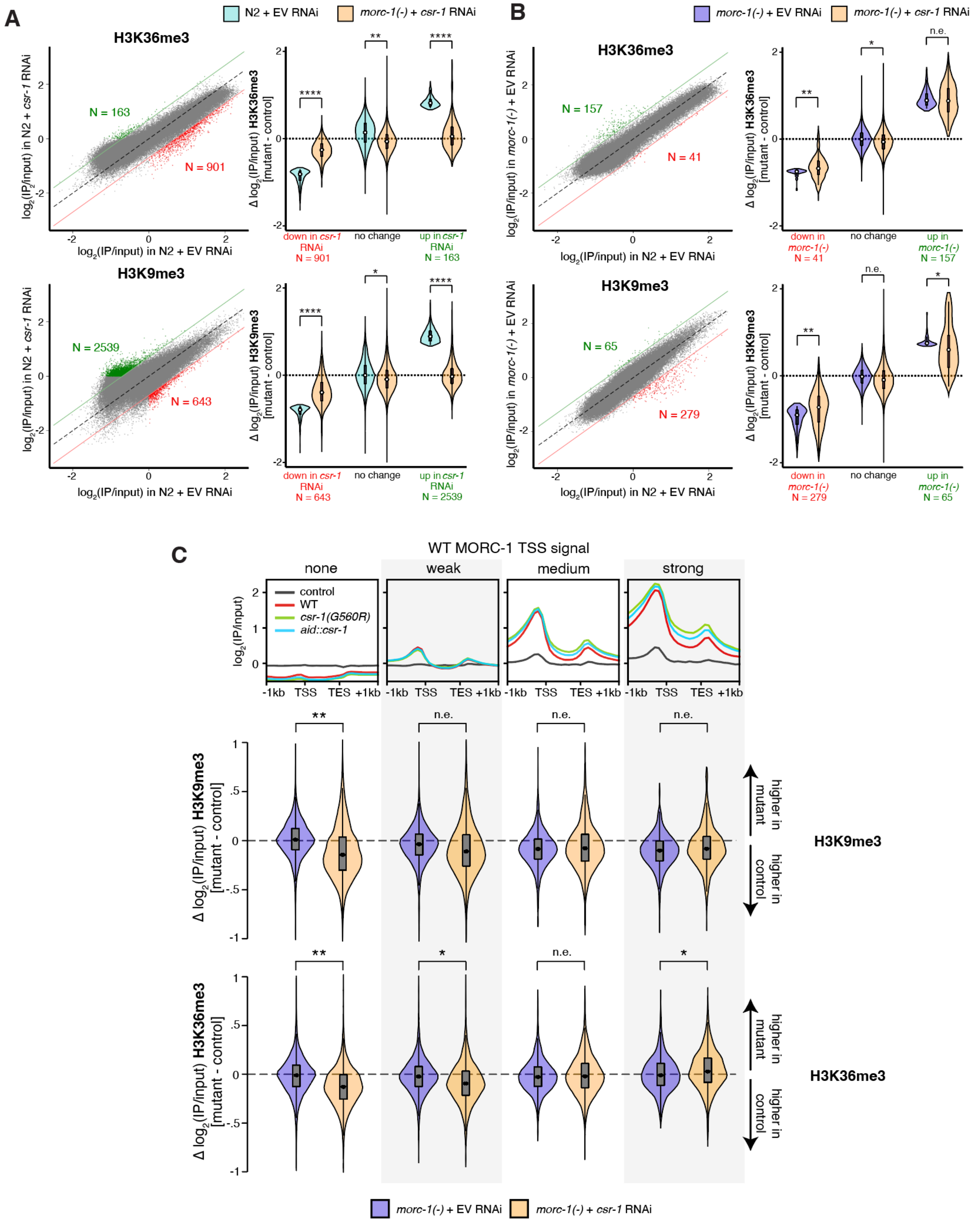

Fig. S15. *morc-1(-)* can rescue chromatin defects in *csr-1* RNAi but not vice-versa. (A–B) Scatterplots same as fig. S12, and identify bins where H3K9me3/H3K36me3 have gone up (red) or down (green) in indicated genotype + RNAi compared to control (wild-type + empty vector (EV) RNAi). In the violin plots, the difference in $\log_2$(IP/input) ChIP-seq signal between indicated

genotype + RNAi and the control is plotted separately for these up, down, and unchanged bins, where y = 0 represents no difference from the control. (A) demonstrates nearly complete rescue of H3K9me3/H3K36me3 changes in *csr-1* RNAi by *morc-1(-)*, while (B) demonstrates lack of rescue of *morc-1(-)* phenotype by *csr-1* RNAi. Significance testing: **** = $p < 0.0001$, *** = $p < 0.001$, ** = $p < 0.01$, * = $p < 0.05$, n.s. = not significant, two-tailed t-test. (**C**) (Top) same as Fig. 2E: metaplot of MORC-1::3xFlag ChIP-seq signal in wild-type germline, *csr-1(G560R)* and *aid::csr-1*, over genes binned by wild-type MORC-1 levels at the TSS. (Bottom) Distribution of change in H3K9me3 and H3K36me3 in *morc-1(-)* treated with EV RNAi or *csr-1* RNAi, compared to control (N2 treated with EV RNAi), over genes binned by wild-type MORC-1 levels at the TSS (same as top panel). Effect size measured using Cohen's d. n.e. = no/minimal effect ($|d| < 0.2$), * = $|d| > 0.2$, ** = $|d| > 0.5$. For all comparisons, $p \sim 0$ by Student's t-test or similar, due to large sample size.

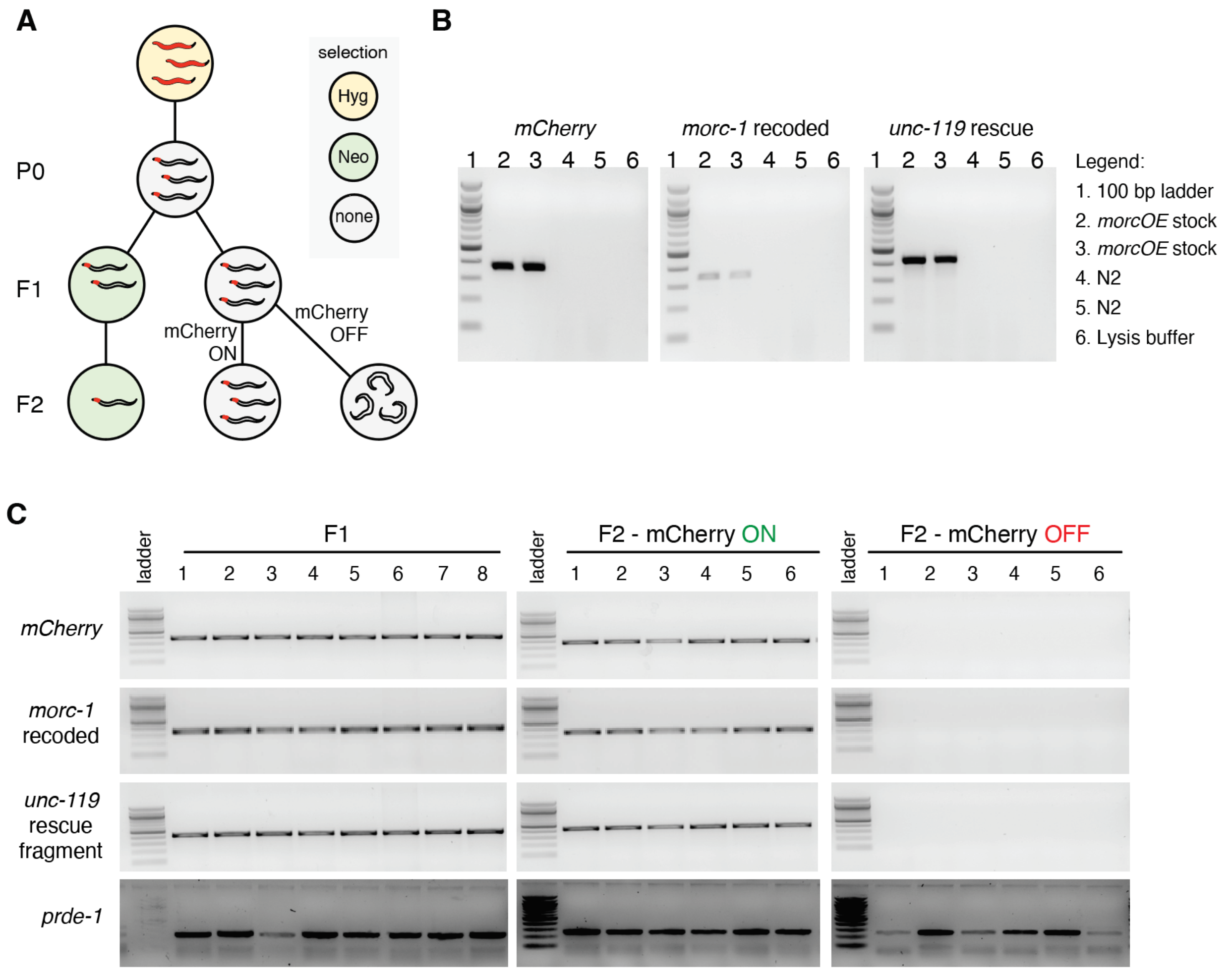

Fig. S16. *morc-1OE* worms that lose mCherry signal also lose the integrated transgene. (**A**) Schematic of experimental design (same as Fig. 5D). **(B)** DNA gels of either *morcOE* stock worms or N2 worms genotyped for presence of (left) mCherry, (middle) the codon-optimized *morc-1* transgene (*morc-1* recoded), and (right) the *unc-119* rescue fragment. **(C)** DNA gels of *morcOE* worms genotyped for presence of mCherry, the codon-optimized *morc-1* transgene (*morc-1* recoded), the *unc-119* rescue fragment, and *prde-1*, an endogenous gene serving as a positive control. Worms were genotyped at the F1 generation (left), and then their progeny, the F2 generation, were separated based on the presence (middle) or absence (right) of visible mCherry signal and then genotyped.

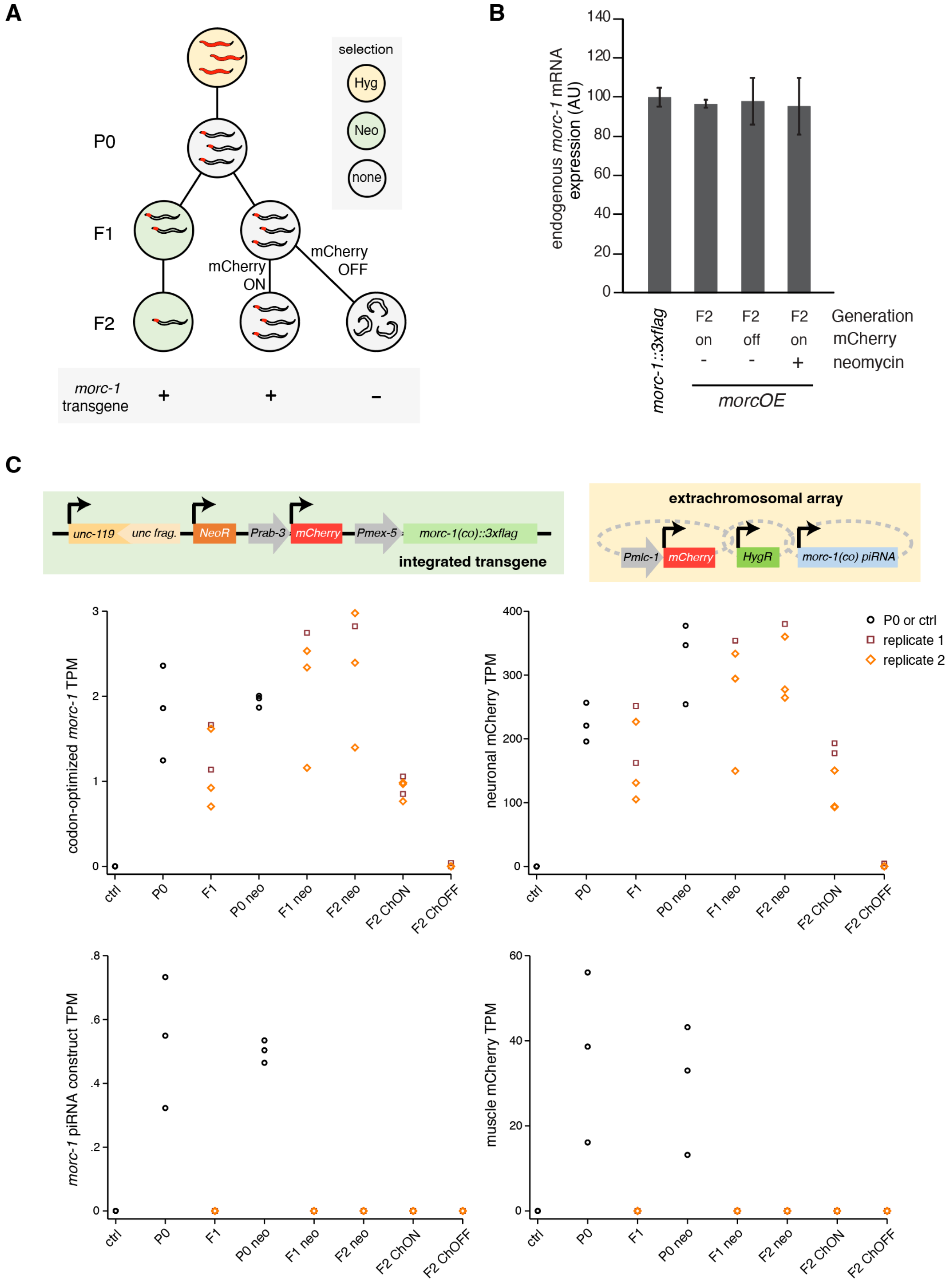

Fig. S17. Expression of genes on integrated *morc-1* transgene and extrachromosomal array in *morcOE* samples. (**A**) Schematic of experimental design (same as Fig. 5D). (**B**) mRNA qPCR

for the endogenous *morc-1* transcript in F2 *morcOE* worms vs. a non-transgenic control (*morc-1::3xflag* CRISPR strain). Error bars represent standard deviation of two technical replicates (**C**) TPM estimates from each RNA-seq replicate for codon-optimized MORC-1 and neuronal mCherry (both on integrated transgene), and the piRNAi construct and muscle mCherry (both on extrachromosomal array). P0 worms were split into two sets of replicates, which were taken through the experiment in parallel and are labelled here as ‘replicate 1’ and ‘replicate 2’. These are considered biological replicates in all analyses. Diagram of transgene and extrachromosomal array at top for reference. Control is same as (B), an endogenous *morc-1::3xflag* strain that lacks the integrated *morcOE* transgene and piRNAi extrachromosomal array. ChON = neuronal mCherry detected, ChOFF = neuronal mCherry not detected (see Fig. 5B, methods).

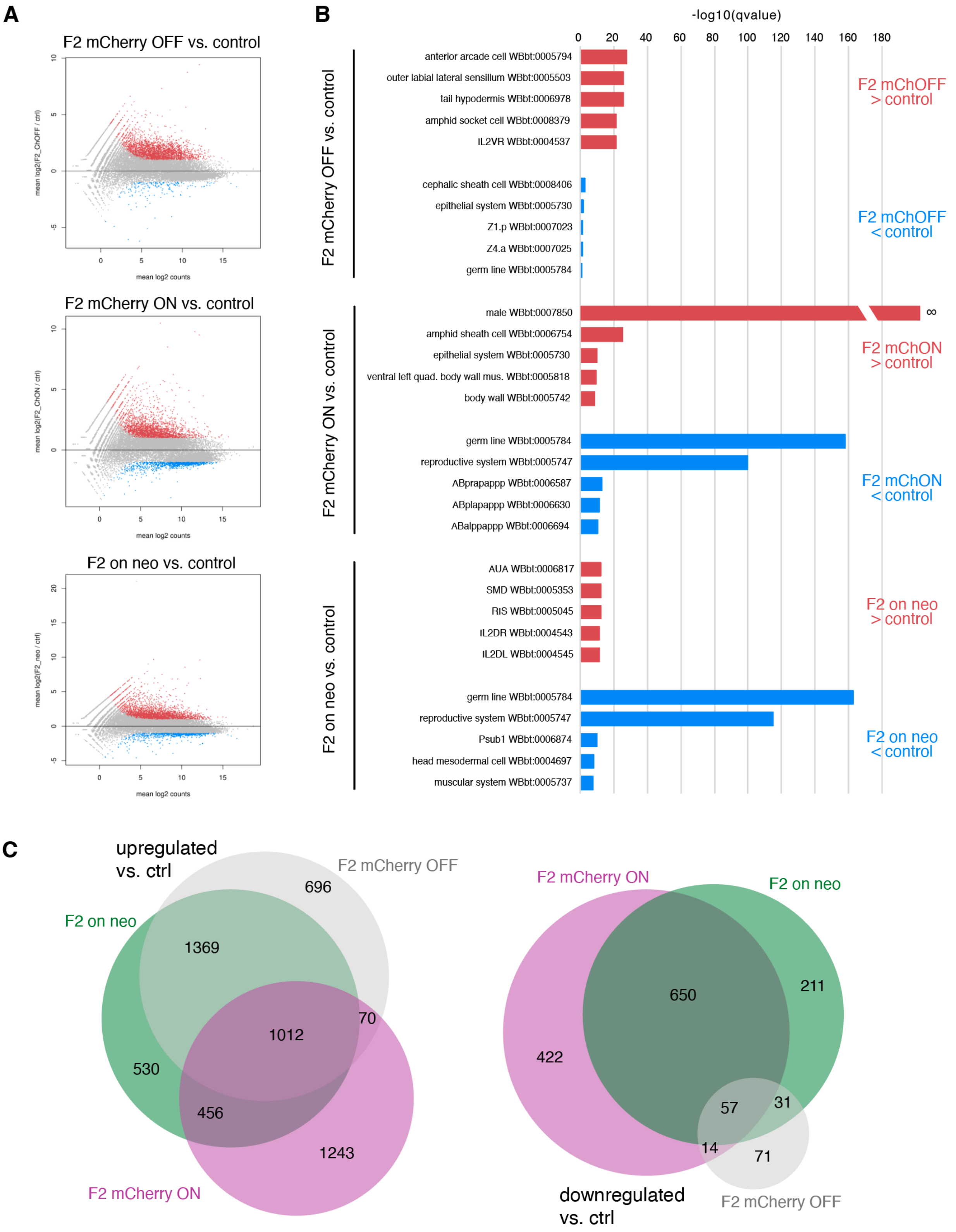

Fig. S18. Characterization of genes up and downregulated in *morcOE* worms. (**A**) DESeq2 (*65*) MA plots for F2 *morcOE* worms expressing the transgene (F2 mCherry ON and F2 on neo) or those that have lost the transgene (F2 mCherry OFF) vs. non-transgenic control (endogenous

morc-1::3xflag only). (**B**) Q-values from tissue enrichment analysis (WormBase, see methods) for genes upregulated or downregulated in each group vs. the non-transgenic control. One term (F2 mCherry ON > ctrl, enrichment term "male") had a q-value of 0, so the corresponding log10(qval) is infinity. (**C**) Overlap between genes upregulated in each condition (left) and downregulated in each condition (right) compared to the non-transgenic control.

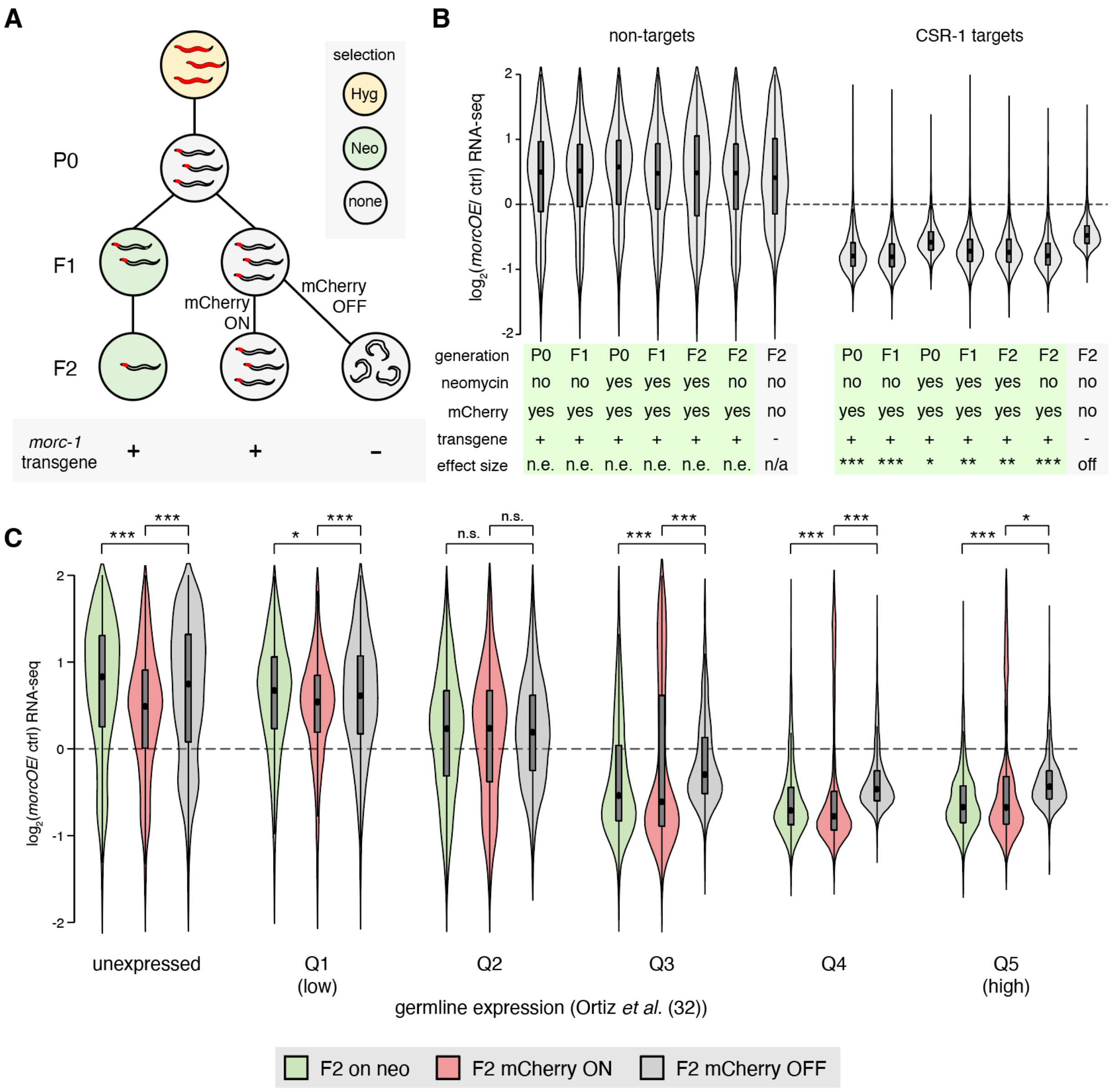

Fig. S19. MORC-1 overexpression in wild-type germline downregulates germline-expressed genes and CSR-1 targets. (**A**) Diagram of experimental design, same as Fig. 5D. (**B**) Distribution of expression changes in indicated sample, across CSR-1 targets (right) and non-targets (left). Bottom row indicates effect size compared to the F2 mCherry OFF sample (grey, right), measured by Cohen's D, n.e. = no/minimal effect ($|d| < 0.2$), * = $|d| > 0.2$, ** = $|d| > 0.5$, *** = $|d| > 0.9$. mCherry = neuronal mCherry, transgene + = transgene present and expressed, - = transgene lost. (**C**) Distribution of expression changes in F2 samples that express the integrated *morc-1* transgene (F2 on neo and F2 mCherry ON) vs. those that have lost the transgene (F2 mCherry OFF), across genes binned according to their expression in the germline in Ortiz *et al.* (*32*). n.s. = not significant, * $p < 0.01$, ** $p < 0.001$, *** $p < 0.0001$, two-tailed t-test.

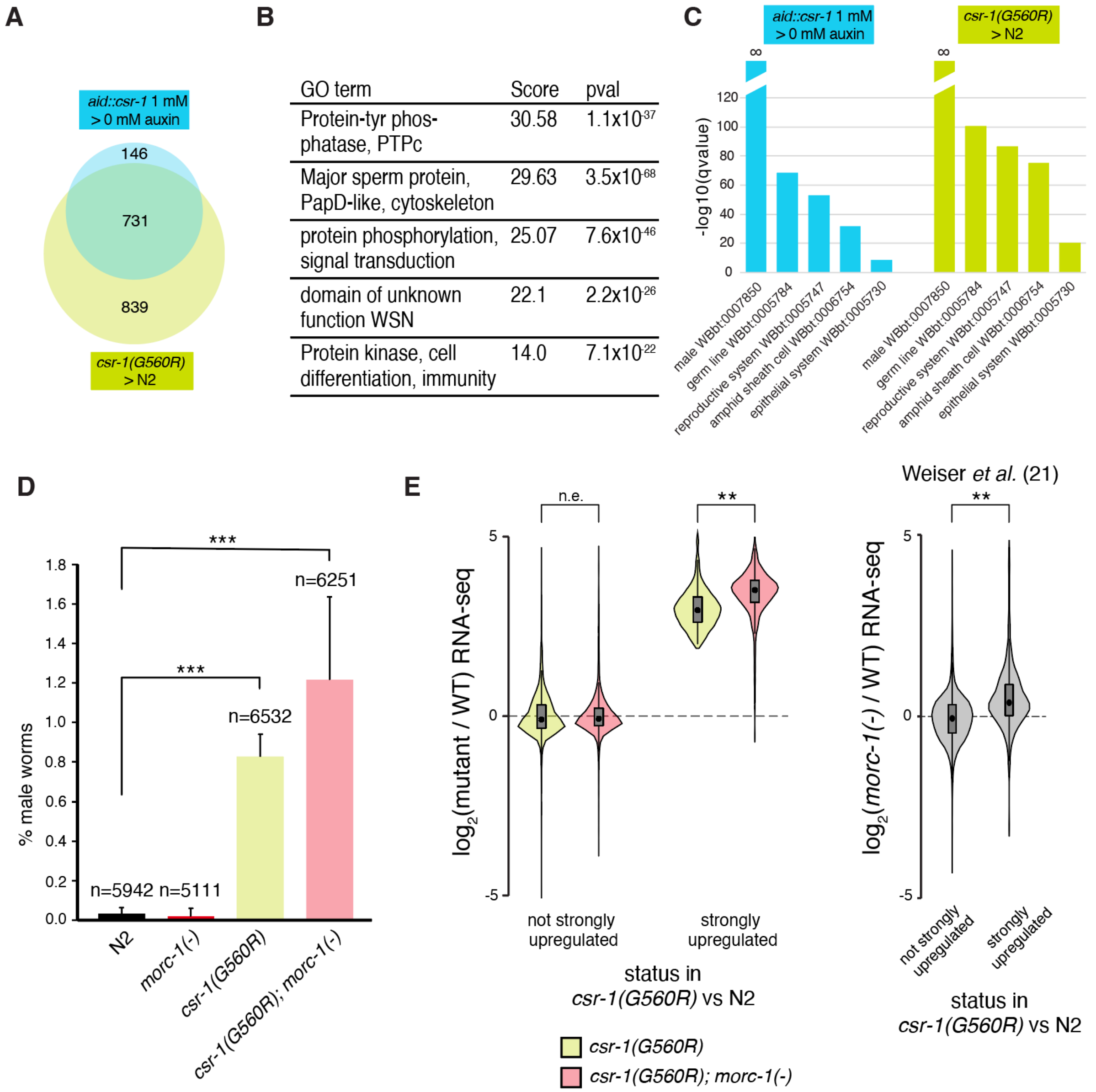

Fig. S20. Him phenotype in *csr-1(G560R)* causes upregulation of sperm-related genes and is not rescued by *morc-1(-)*. (**A**) Overlap between genes significantly upregulated in *csr-1(G560R)* vs. those significantly upregulated in *aid::csr-1* ($\log_2$ fold change > 1 & adjusted p-value < 0.01). (**B**) Top 5 GO-terms for genes significantly upregulated in *csr-1(G560R)* vs. WT ($\log_2$ fold change > 1 & adjusted p-value < 0.01) (DAVID (*67*), see methods). (**C**) Top 5 tissue terms enriched among genes significantly upregulated in *csr-1(G560R)* or *aid::csr-1*, WormBase enrichment analysis (*68*). For two terms, the *q*-value estimate is 0, so -log(*q*) is infinity. (**D**) Percent of male worms among indicated number of progeny from wild-type (N2), *morc-1(-)*, *csr-1(G560R)* and *csr-1(G560R); morc-1(-)* worms. Error bars = standard deviation from 3 independent experiments. *** = p < 0.001, t-test. (**E**) Expression changes in (left) *csr-1(G560R)* and *morc-1(-); csr-1(G560R)* vs. WT (this study) and (right) *morc-1(-)* vs. WT (Weiser et al. (*21*)), over n=1258 genes strongly upregulated in *csr-1(G560R)* compared to WT ($\log_2$ fold change > 2 & adjusted p-value < 0.01), showing that *morc-1(-)* mildly amplifies this upregulation both in wild-type and in *csr-1(G560R)*.

Effect size measured by Cohen’s D, n.e. = no/minimal effect (|d| < 0.2), * = |d| > 0.2, ** = |d| > 0.5, *** = |d| > 0.9, **** = |d| > 1.5.

	Genotype	Description
1	*kstSi74[unc-119(p1+p2, loxP)(+), pmex-5::morc-1::tbb-2]I; unc-119(ed3)III*	*morc-1* insertion into Chr I - single copy insertion
2	*kstSi75[unc-119(p1+p2, loxP)(+), pmex-5::morc-1::tbb-2]I; unc-119(ed3)III*	*morc-1* insertion into Chr I - single copy insertion
3	*kstSi76[unc-119(p1+p2, loxP)(+), pmex-5::morc-1::tbb-2, pmlc-1::mCherry::NLS]I; unc-119(ed3)III*	*morc-1* insertion into Chr I - complex insertion
4	*kstSi82[unc-119(p1+p2, loxP)(+), pmex-5::morc-1::tbb-2]I; unc-119(ed3)III*	*morc-1* insertion into Chr I - single copy insertion
5	*kstSi77[unc-119(p1+p2, loxP)(+), pmex-5::morc-1::tbb-2]II; unc-119(ed3)III*	*morc-1* insertion into Chr II - single copy insertion
6	*kstSi78[unc-119(p1+p2, loxP)(+), pmex-5::morc-1::tbb-2]II; unc-119(ed3)III*	*morc-1* insertion into Chr II - single copy insertion
7	*kstSi79[unc-119(p1+p2, loxP)(+), pmex-5::morc-1::tbb-2]II; unc-119(ed3)III*	*morc-1* insertion into Chr II - single copy insertion
8	*kstSi80[unc-119(p1+p2, loxP)(+), pmex-5::morc-1::tbb-2]II; unc-119(ed3)III*	*morc-1* insertion into Chr II - single copy insertion
9	*unc-119(ed3)III; kstSi81[unc-119(p1+p2, loxP)(+), pmex-5::morc-1::tbb-2]IV*	*morc-1* insertion into Chr IV - complex insertion
10	*kstSi32[unc-119(p1, spc2)(-)] I; unc-119(ed3)III; kstEx44[pEXP[4-3][Phsp-16.41 (no start) _ Cas9 (start, stop, syntrons) _gpd-2 TagRFP-T(start, stop, PATCs, myr) smu-1 UTR, rpr-1::spacer 2, pmex-5::morc-1::tbb-2, Pmlc-1::mCherry::NSL, HygroR]*	extrachromosomal array
11	*pmorc-1(short)::morc-1::3xflag::morc-1* (0.5 ng/uL)	integrated array
12	*ppie-1::morc-1::3xflag::morc-1* (5ng/uL)	integrated array
13	*ppie-1::morc-1::3xflag::morc-1* (50ng/uL)	integrated array
14	*pmorc-1(long)::gfp::morc-1::3xflag::morc-1* (5ng/uL)	integrated array
15	*pmex-5::CO-morc-1::3xflag::tbb-2* (5ng/uL)	extrachromosomal array containing codon-optimized *morc-1*
16	*pmex-5::CO-morc-1::3xflag::tbb-2* (20ng/uL)	extrachromosomal array containing codon-optimized *morc-1*
17	*pmex-5::CO-morc-1::3xflag::tbb-2* (32ng/uL)	extrachromosomal array containing codon-optimized *morc-1*
18	*pmex-5::CO-morc-1::3xflag::tbb-2* (40ng/uL)	extrachromosomal array containing codon-optimized *morc-1*
19	*pmex-5::CO-morc-1::3xflag::tbb-2* (50ng/uL)	extrachromosomal array containing codon-optimized *morc-1*
20	*pmex-5::gfp::morc-1::3xflag::tbb-2* (50ng/uL)	extrachromosomal array containing codon-optimized *morc-1*
21	*kstSi96[pmex-5::morc-1PATC::gpd2::cegfp.::tbb-2] II*	*morc-1* (with PATCs) insertion into Chr II - Single copy insertion
22	*kstSi96[pmex-5::morc-1PATC::gpd2::cegfp.::tbb-2] II*	*morc-1* (with PATCs) insertion into Chr II - Single copy insertion
23	*kstSi96[pmex-5::morc-1PATC::gpd2::cegfp.::tbb-2] II*	*morc-1* (with PATCs) insertion into Chr II - Single copy insertion

Table S1. Failed strategies for generating a MORC-1 overexpressing line. Lists all constructs and strategies we attempted to generate a strain to overexpress *morc-1* in the germline, from which we were not able to recover worms overexpressing *morc-1* due to sterility.

	Seq Type	Genotype	ChIP	Tissue	SRA/Encode ID(s)	Ref
1	ChIP-chip	N2	H3K4me3	whole worms	Encode dataset: 3552	(*73*)
2	ChIP-seq	N2	H3K27ac	germline	SRR9214969	(*31*)
3	ChIP-seq	N2	H3K27ac	germline	SRR9214971	(*31*)
4	ChIP-seq	N2	input	germline	SRR9214970	(*31*)
5	ChIP-seq	N2	input	germline	SRR9214972	(*31*)
6	ChIP-seq	N2	H3K36me3	whole worms	SRR5024048	(*21*)
7	ChIP-seq	N2	H3K36me3	whole worms	SRR5024049	(*21*)
8	ChIP-seq	N2	H3K36me3	whole worms	SRR5024050	(*21*)
9	ChIP-seq	*morc-1(-)*	input	whole worms	SRR5024036	(*21*)
10	ChIP-seq	*morc-1(-)*	H3K36me3	whole worms	SRR5024052	(*21*)
11	ChIP-seq	*morc-1(-)*	H3K36me3	whole worms	SRR5024053	(*21*)
12	ChIP-seq	N2	input	whole worms	SRR5024038	(*21*)
13	ChIP-seq	*morc-1(-)*	H3K9me3	whole worms	SRR5024028	(*21*)
14	ChIP-seq	*morc-1(-)*	H3K9me3	whole worms	SRR5024030	(*21*)
15	ChIP-seq	N2	H3K9me3	whole worms	SRR5024031	(*21*)
16	RNA-seq	N2	n/a	whole worms	SRR5024017	(*21*)
17	RNA-seq	N2	n/a	whole worms	SRR5024018	(*21*)
18	RNA-seq	*morc-1(-)*	n/a	whole worms	SRR5024021	(*21*)
19	RNA-seq	*morc-1(-)*	n/a	whole worms	SRR5024022	(*21*)
20	RNA-seq	*fog-2(-)*	n/a	whole worms	SRR1263137 SRR1263138 SRR1263139 SRR1263140	(*32*)
21	RNA-seq	*fog-2(-)*	n/a	whole worms	SRR1263141 SRR1263142 SRR1263143 SRR1263144	(*32*)
22	RNA-seq	*fog-2(-)*	n/a	whole worms	SRR1263145 SRR1263146 SRR1263147 SRR1263148	(*32*)
23	RNA-seq	*fog-2(-)*	n/a	whole worms	SRR1263149 SRR1263150 SRR1263151 SRR1263152	(*32*)
24	RNA-seq	*fog-2(-)*	n/a	whole worms	SRR1263153 SRR1263154 SRR1263155 SRR1263156	(*32*)
25	RNA-seq	*fog-2(-)*	n/a	whole worms	SRR1263157 SRR1263158 SRR1263159 SRR1263160	(*32*)
26	RNA-seq	*fog-2(-)*	n/a	whole worms	SRR1263161 SRR1263162 SRR1263163 SRR1263164	(*32*)
27	RNA-seq	*fog-2(-)*	n/a	whole worms	SRR1263165 SRR1263166 SRR1263167 SRR1263168	(*32*)
28	RNA-seq	*fem-3(-)*	n/a	whole worms	SRR1263169 SRR1263170 SRR1263171 SRR1263172	(*32*)
29	RNA-seq	*fem-3(-)*	n/a	whole worms	SRR1263173 SRR1263174 SRR1263175 SRR1263176	(*32*)
30	RNA-seq	*fem-3(-)*	n/a	whole worms	SRR1263177 SRR1263178 SRR1263179 SRR1263180	(*32*)
31	RNA-seq	*fem-3(-)*	n/a	whole worms	SRR1263181 SRR1263182 SRR1263183 SRR1263184	(*32*)
32	RNA-seq	*fem-3(-)*	n/a	whole worms	SRR1263185 SRR1263186 SRR1263187 SRR1263188	(*32*)
33	RNA-seq	*fem-3(-)*	n/a	whole worms	SRR1263189 SRR1263190 SRR1263191 SRR1263192	(*32*)
34	RNA-seq	*fem-3(-)*	n/a	whole worms	SRR1263193 SRR1263194 SRR1263195 SRR1263196	(*32*)

35	RNA-seq	*fem-3(-)*	n/a	whole worms	SRR1263197 SRR1263198 SRR1263199 SRR1263200	(*32*)

Table S2. Published sequencing datasets used in this study. N2 = wild-type strain Bristol N2.

Data S1. Mapping statistics for all sequencing data. Number of reads that were obtained from sequencing, that passed QC filtering, that aligned uniquely, and that remained after PCR deduplication, for all libraries generated in this study, as well as all published sequencing datasets reanalyzed for this study using the same pipeline.

Data S2. Full dataset used for analyses over genes. Excel file containing three sheets. The first ("gene data") contains per-gene level RNA-seq, sRNA-seq, RIP-seq, ChIP-seq and ATAC-seq data used to generate most figures in this study. ChIP-seq data were averaged over either gene body or transcriptional start site (TSS) region (see methods). Also includes several other useful variables (flags for CSR-1 targets, published germline expression data, etc.). The second ("transgene data") contains TPM estimates for all *morcOE* samples for expression of genes expressed either from the integrated transgene or from the extrachromosomal array in that line. The last sheet ("list of variables") contains detailed information on each variable in both sheets.

Data S3. H3K9me3 and H3K36me3 ChIP-seq signal over 1-kb bins genome-wide. Average ChIP-seq signal (log_2(IP/input)) across 1 kb bins tiled genome-wide. Includes H3K9me3 and H3K36me3 data (all replicates merged) for N2, N2 + *csr-1* RNAi, *morc-1(-)*, and *morc-1(-)* + *csr-1* RNAi; used for figs. S11, S14. The "variable info" tab contains information on each variable.

Data S4. Fertility & Him assay data. Raw data for all fertility assays and high incidence of males (Him) assays. Fertility assays in F2 *morcOE* worms (Fig. 5C) compare our *morcOE* strain separated into transgene (tg) expressed or lost, vs. a non-transgenic control expressing only *morc-1::3xflag* from the endogenous locus. Status of each parent's movement (Unc or wild-type) and mCherry phenotypes also recorded, as well as inferred *morc-1* transgene status (tg expressed or tg lost). Worms were transferred to new plates as progeny were laid to facilitate counting, and counts on each plate are recorded separately. This data was also collected across two different experiments, referred to as replicate 1 and 2. All other fertility assays (Fig. 1A, 1C, fig. S1E) are recorded in another tab, and only total progeny are listed. For Him assays, the experiment was repeated 3 times.

REFERENCES AND NOTES

1. U. Seroussi, A. Lugowski, L. Wadi, R. X. Lao, A. R. Willis, W. Zhao, A. E. Sundby, A. G. Charlesworth, A. W. Reinke, J. M. Claycomb, A comprehensive survey of *C. elegans* argonaute proteins reveals organism-wide gene regulatory networks and functions *eLife* **12**, e83853 (2023).

2. E. Yigit, P. J. Batista, Y. Bei, K. M. Pang, C.-C. G. Chen, N. H. Tolia, L. Joshua-Tor, S. Mitani, M. J. Simard, C. C. Mello, Analysis of the *C. elegans* Argonaute family reveals that distinct Argonautes act sequentially during RNAi. *Cell* **127**, 747–757 (2006).

3. J. M. Claycomb, P. J. Batista, K. M. Pang, W. Gu, J. J. Vasale, J. C. van Wolfswinkel, D. A. Chaves, M. Shirayama, S. Mitani, R. F. Ketting, D. Conte, C. C. Mello, The Argonaute CSR-1 and its 22G-RNA cofactors are required for holocentric chromosome segregation. *Cell* **139**, 123–134 (2009).

4. G. Cecere, S. Hoersch, S. O'Keeffe, R. Sachidanandam, A. Grishok, Global effects of the CSR-1 RNA interference pathway on the transcriptional landscape. *Nat. Struct. Mol. Biol.* **21**, 358–365 (2014).

5. D. L. Updike, S. Strome, A genomewide RNAi screen for genes that affect the stability, distribution and function of P granules in *Caenorhabditis elegans*. *Genetics* **183**, 1397–1419 (2009).

6. A. C. Campbell, D. L. Updike, CSR-1 and P granules suppress sperm-specific transcription in the *C. elegans* germline *Development* **142**, 1745–1755 (2015).

7. D. C. Avgousti, S. Palani, Y. Sherman, A. Grishok, CSR-1 RNAi pathway positively regulates histone expression in *C. elegans*. *EMBO J.* **31**, 3821–3832 (2012).

8. M. Seth, M. Shirayama, W. Gu, T. Ishidate, D. Conte Jr., C. C. Mello, The *C. elegans CSR-1* argonaute pathway counteracts epigenetic silencing to promote germline gene expression. *Dev. Cell* **27**, 656–663 (2013).

9. C. J. Wedeles, M. Z. Wu, J. M. Claycomb, Protection of germline gene expression by the *C. elegans* Argonaute CSR-1. *Dev. Cell* **27**, 664–671 (2013).

10. E. Cornes, L. Bourdon, M. Singh, F. Mueller, P. Quarato, E. Wernersson, M. Bienko, B. Li, G. Cecere, piRNAs initiate transcriptional silencing of spermatogenic genes during *C. elegans* germline development. *Dev. Cell* **57**, 180–196.e7 (2022).

11. W.-S. Wu, J. S. Brown, S.-C. Shiue, C.-J. Chung, D.-E. Lee, D. Zhang, H.-C. Lee, Transcriptome-wide analyses of piRNA binding sites suggest distinct mechanisms regulate piRNA binding and silencing in *C. elegans RNA* **29**, 557–569 (2023).

12. M. Singh, E. Cornes, B. Li, P. Quarato, L. Bourdon, F. Dingli, D. Loew, S. Proccacia, G. Cecere, Translation and codon usage regulate Argonaute slicer activity to trigger small RNA biogenesis. *Nat. Commun.* **12**, 3492 (2021).

13. K. Aoki, H. Moriguchi, T. Yoshioka, K. Okawa, H. Tabara, In vitro analyses of the production and activity of secondary small interfering RNAs in C. elegans. *EMBO J.* **26**, 5007–5019 (2007).

14. A. Gerson-Gurwitz, S. Wang, S. Sathe, R. Green, G. W. Yeo, K. Oegema, A. Desai, A small RNA-catalytic Argonaute pathway tunes germline transcript levels to ensure embryonic divisions. *Cell* **165**, 396–409 (2016).

15. K. Friend, Z. T. Campbell, A. Cooke, P. Kroll-Conner, M. P. Wickens, J. Kimble, A conserved PUF–Ago–eEF1A complex attenuates translation elongation. *Nat. Struct. Mol. Biol.* **19**, 176–183 (2012).

16. A. M. Kershner, J. Kimble, Genome-wide analysis of mRNA targets for *Caenorhabditis elegans* FBF, a conserved stem cell regulator. *Proc. Natl. Acad. Sci. U.S.A.* **107**, 3936–3941 (2010).

17. G. Moissiard, S. J. Cokus, J. Cary, S. Feng, A. C. Billi, H. Stroud, D. Husmann, Y. Zhan, B. R. Lajoie, R. P. McCord, C. J. Hale, W. Feng, S. D. Michaels, A. R. Frand, M. Pellegrini, J. Dekker, J. K. Kim, S. E. Jacobsen, MORC family ATPases required for heterochromatin condensation and gene silencing. *Science* **336**, 1448–1451 (2012).

18. W. A. Pastor, H. Stroud, K. Nee, W. Liu, D. Pezic, S. Manakov, S. A. Lee, G. Moissiard, N. Zamudio, D. Bourc'his, A. A. Aravin, A. T. Clark, S. E. Jacobsen, MORC1 represses transposable elements in the mouse male germline. *Nat. Commun.* **5**, 5795 (2014).

19. C. J. Harris, D. Husmann, W. Liu, F. E. Kasmi, H. Wang, A. Papikian, W. A. Pastor, G. Moissiard, A. A. Vashisht, J. L. Dangl, J. A. Wohlschlegel, S. E. Jacobsen, Arabidopsis AtMORC4 and AtMORC7 form nuclear bodies and repress a large number of protein-coding genes. *PLOS Genet.* **12**, e1005998 (2016).

20. I. A. Tchasovnikarova, R. T. Timms, C. H. Douse, R. C. Roberts, G. Dougan, R. E. Kingston, Y. Modis, P. J. Lehner, Hyperactivation of HUSH complex function by Charcot–Marie–Tooth disease mutation in *MORC2*. *Nat. Genet.* **49**, 1035–1044 (2017).

21. N. E. Weiser, D. X. Yang, S. Feng, N. Kalinava, K. C. Brown, J. Khanikar, M. A. Freeberg, M. J. Snyder, G. Csankovszki, R. C. Chan, S. G. Gu, T. A. Montgomery, S. E. Jacobsen, J. K. Kim, MORC-1 integrates nuclear RNAi and transgenerational chromatin architecture to promote germline immortality. *Dev. Cell* **41**, 408–423.e7 (2017).

22. H. Kim, L. Yen, S. P. Wongpalee, J. A. Kirshner, N. Mehta, Y. Xue, J. B. Johnston, A. L. Burlingame, J. K. Kim, J. J. Loparo, S. E. Jacobsen, The gene-silencing protein MORC-1 topologically entraps DNA and forms multimeric assemblies to cause DNA compaction. *Mol. Cell* **75**, 700–710.e6 (2019).

23. D. C. Farhat, C. Swale, C. Dard, D. Cannella, P. Ortet, M. Barakat, F. Sindikubwabo, L. Belmudes, P.-J. De Bock, Y. Couté, A. Bougdour, M.-A. Hakimi, A MORC-driven transcriptional switch controls Toxoplasma developmental trajectories and sexual commitment. *Nat. Microbiol.* **5**, 570–583 (2020).

24. Y. Xue, Z. Zhong, C. J. Harris, J. Gallego-Bartolomé, M. Wang, C. Picard, X. Cao, S. Hua, I. Kwok, S. Feng, Y. Jami-Alahmadi, J. Sha, J. Gardiner, J. Wohlschlegel, S. E. Jacobsen, Arabidopsis MORC proteins function in the efficient establishment of RNA directed DNA methylation. *Nat. Commun.* **12**, 4292 (2021).

25. V. P. Desai, J. Chouaref, H. Wu, W. A. Pastor, R. L. Kan, H. M. Oey, Z. Li, J. Ho, K. K. D. Vonk, D. S. L. Granado, M. A. Christopher, A. T. Clark, S. E. Jacobsen, L. Daxinger, The role of MORC3 in silencing transposable elements in mouse embryonic stem cells. *Epigenetics Chromatin* **14**, 49 (2021).

26. Y. Shao, Y. Li, J. Zhang, D. Liu, F. Liu, Y. Zhao, T. Shen, F. Li, Involvement of histone deacetylation in MORC2-mediated down-regulation of carbonic anhydrase IX. *Nucleic Acids Res.* **38**, 2813–2824 (2010).

27. Q. Zhang, Y. Song, W. Chen, X. Wang, Z. Miao, L. Cao, F. Li, G. Wang, By recruiting HDAC1, MORC2 suppresses p21Waf1/Cip1 in gastric cancer. *Oncotarget* **6**, 16461–16470 (2015).

28. M. L. Watson, A. R. Zinn, N. Inoue, K. D. Hess, J. Cobb, M. A. Handel, R. Halaban, C. C. Duchene, G. M. Albright, R. W. Moreadith, Identification of *morc* (*microrchidia*), a mutation that results in arrest of spermatogenesis at an early meiotic stage in the mouse *Proc. Natl. Acad. Sci. U.S.A.* **95**, 14361–14366 (1998).

29. A. Y. Zinovyeva, I. Veksler-Lublinsky, A. A. Vashisht, J. A. Wohlschlegel, V. R. Ambros, *Caenorhabditis elegans* ALG-1 antimorphic mutations uncover functions for Argonaute in microRNA guide strand selection and passenger strand disposal. *Proc. Natl. Acad. Sci. U.S.A.* **112**, E5271–E5280 (2015).

30. L. Zhang, J. D. Ward, Z. Cheng, A. F. Dernburg, The auxin-inducible degradation (AID) system enables versatile conditional protein depletion in *C. elegans*. *Development* **142**, 4374–4384 (2015).

31. M. Han, G. Wei, C. E. McManus, L. W. Hillier, V. Reinke, Isolated *C. elegans* germ nuclei exhibit distinct genomic profiles of histone modification and gene expression. *BMC Genomics* **20**, 500 (2019).

32. M. A. Ortiz, D. Noble, E. P. Sorokin, J. Kimble, A new dataset of spermatogenic vs. oogenic transcriptomes in the nematode *Caenorhabditis elegans*. *G3* **4**, 1765–1772 (2014).

33. E. S. Gushchanskaia, R. Esse, Q. Ma, N. C. Lau, A. Grishok, Interplay between small RNA pathways shapes chromatin landscapes in *C. elegans*. *Nucleic Acids Res.* **47**, 5603–5616 (2019).

34. S. E. Mouridi, F. Alkhaldi, C. Frøkjær-Jensen, Modular safe-harbor transgene insertion for targeted single-copy and extrachromosomal array integration in *Caenorhabditis elegans*. *G3* **12**, jkac184 (2022).

35. M. Priyadarshini, J. Z. Ni, A. M. Vargas-Velazquez, S. G. Gu, C. Frøkjær-Jensen, Reprogramming the piRNA pathway for multiplexed and transgenerational gene silencing in *C. elegans*. *Nat. Methods* **19**, 187–194 (2022).

36. P. Quarato, M. Singh, E. Cornes, B. Li, L. Bourdon, F. Mueller, C. Didier, G. Cecere, Germline inherited small RNAs facilitate the clearance of untranslated maternal mRNAs in *C. elegans* embryos. *Nat. Commun.* **12**, 1441 (2021).

37. F. H. Andrews, Q. Tong, K. D. Sullivan, E. M. Cornett, Y. Zhang, M. Ali, J. Ahn, A. Pandey, A. H. Guo, B. D. Strahl, J. C. Costello, J. M. Espinosa, S. B. Rothbart, T. G. Kutateladze, Multivalent chromatin engagement and inter-domain crosstalk regulate MORC3 ATPase. *Cell Rep.* **16**, 3195–3207 (2016).

38. S. Li, L. Yen, W. A. Pastor, J. B. Johnston, J. Du, C. J. Shew, W. Liu, J. Ho, B. Stender, A. T. Clark, A. L. Burlingame, L. Daxinger, D. J. Patel, S. E. Jacobsen, Mouse MORC3 is a GHKL ATPase that localizes to H3K4me3 marked chromatin *Proc. Natl. Acad. Sci. U.S.A.* **113**, E5108–E5116 (2016).

39. A. H. Tencer, K. L. Cox, G. M. Wright, Y. Zhang, C. J. Petell, B. J. Klein, B. D. Strahl, J. C. Black, M. G. Poirier, T. G. Kutateladze, Molecular mechanism of the MORC4 ATPase activation. *Nat. Commun.* **11**, 5466 (2020).

40. Y. Liu, W. Tempel, Q. Zhang, X. Liang, P. Loppnau, S. Qin, J. Min, Family-wide characterization of histone binding abilities of human CW domain-containing proteins. *J. Biol. Chem.* **291**, 9000–9013 (2016).

41. H. Wang, L. Zhang, Q. Luo, J. Liu, G. Wang, MORC protein family-related signature within human disease and cancer. *Cell Death Dis.* **12**, 1112 (2021).

42. Z. Zhong, Y. Xue, C. J. Harris, M. Wang, Z. Li, Y. Ke, M. Liu, J. Zhou, Y. Jami-Alahmadi, S. Feng, J. A. Wohlschlegel, S. E. Jacobsen, MORC proteins regulate transcription factor binding by mediating chromatin compaction in active chromatin regions. *Genome Biol.* **24**, 96 (2023).

43. M. Liu, X. Sun, S. Shi, MORC2 enhances tumor growth by promoting angiogenesis and tumor-associated macrophage recruitment via Wnt/β-catenin in lung cancer. *Cell. Physiol. Biochem.* **51**, 1679–1694 (2018).

44. J. Liu, Y. Shao, Y. He, K. Ning, X. Cui, F. Liu, Z. Wang, F. Li, MORC2 promotes development of an aggressive colorectal cancer phenotype through inhibition of NDRG1. *Cancer Sci.* **110**, 135–146 (2019).

45. Z. Pan, Q. Ding, Q. Guo, Y. Guo, L. Wu, L. Wu, M. Tang, H. Yu, F. Zhou, MORC2, a novel oncogene, is upregulated in liver cancer and contributes to proliferation, metastasis and chemoresistance. *Int. J. Oncol.* **53**, 59–72 (2018).

46. S. Zhang, A. Guo, H. Wang, J. Liu, C. Dong, J. Ren, G. Wang, Oncogenic MORC2 in cancer development and beyond. *Genes Dis.* **11**, 861–873 (2024).

47. S. Brenner, The genetics of *Caenorhabditis elegans*. *Genetics* **77**, 71–94 (1974).

48. A. Paix, A. Folkmann, D. Rasoloson, G. Seydoux, High efficiency, homology-directed genome editing in *Caenorhabditis elegans* using CRISPR-Cas9 ribonucleoprotein complexes. *Genetics* **201**, 47–54 (2015).

49. R. S. Kamath, J. Ahringer, Genome-wide RNAi screening in *Caenorhabditis elegans*. *Methods* **30**, 313–321 (2003).

50. L. Timmons, A. Fire, Specific interference by ingested dsRNA. *Nature* **395**, 854 (1998).

51. E. Zanin, J. Dumont, R. Gassmann, I. Cheeseman, P. Maddox, S. Bahmanyar, A. Carvalho, S. Niessen, J. R Yates III, K. Oegema, A. Desai, Chapter 11 - Affinity purification of protein complexes in *C. elegans*. *Methods Cell Biol.* **106**, 289–322 (2011).

52. Z. Zhong, S. Feng, S. H. Duttke, M. E. Potok, Y. Zhang, J. Gallego-Bartolomé, W. Liu, S. E. Jacobsen, DNA methylation-linked chromatin accessibility affects genomic architecture in *Arabidopsis*. *Proc. Natl. Acad. Sci. U.S.A.* **118**, e2023347118 (2021).

53. S. Andrews, FastQC: A Quality Control tool for High Throughput Sequence Data (2010). https://www.bioinformatics.babraham.ac.uk/projects/fastqc/.

54. F. Krueger, Trim Galore (2012). https://www.bioinformatics.babraham.ac.uk/projects/trim_galore/.

55. A. Dobin, C. A. Davis, F. Schlesinger, J. Drenkow, C. Zaleski, S. Jha, P. Batut, M. Chaisson, T. R. Gingeras, STAR: Ultrafast universal RNA-seq aligner. *Bioinformatics* **29**, 15–21 (2013).

56. B. Langmead, S. L. Salzberg, Fast gapped-read alignment with Bowtie 2. *Nat. Methods* **9**, 357–359 (2012).

57. Picard toolkit (2019). https://github.com/broadinstitute/picard.

58. J. M. Gaspar, GitHub - jsh58/Genrich: Detecting sites of genomic enrichment (2018). https://github.com/jsh58/Genrich.

59. F. Ramírez, D. P. Ryan, B. Grüning, V. Bhardwaj, F. Kilpert, A. S. Richter, S. Heyne, F. Dündar, T. Manke, deepTools2: A next generation web server for deep-sequencing data analysis. *Nucleic Acids Res.* **44**, W160–W165 (2016).

60. P. V. Kharchenko, M. Y. Tolstorukov, P. J. Park, Design and analysis of ChIP-seq experiments for DNA-binding proteins. *Nat. Biotechnol.* **26**, 1351–1359 (2008).

61. S. G. Landt, G. K. Marinov, A. Kundaje, P. Kheradpour, F. Pauli, S. Batzoglou, B. E. Bernstein, P. Bickel, J. B. Brown, P. Cayting, Y. Chen, G. DeSalvo, C. Epstein, K. I. Fisher-Aylor, G.

Euskirchen, M. Gerstein, J. Gertz, A. J. Hartemink, M. M. Hoffman, V. R. Iyer, Y. L. Jung, S. Karmakar, M. Kellis, P. V. Kharchenko, Q. Li, T. Liu, X. S. Liu, L. Ma, A. Milosavljevic, R. M. Myers, P. J. Park, M. J. Pazin, M. D. Perry, D. Raha, T. E. Reddy, J. Rozowsky, N. Shoresh, A. Sidow, M. Slattery, J. A. Stamatoyannopoulos, M. Y. Tolstorukov, K. P. White, S. Xi, P. J. Farnham, J. D. Lieb, B. J. Wold, M. Snyder, ChIP-seq guidelines and practices of the ENCODE and modENCODE consortia. *Genome Res.* **22**, 1813–1831 (2012).

62. J. M. Gaspar, Improved peak-calling with MACS2. bioRxiv 496521 [Preprint] (2018). https://doi.org/10.1101/496521.

63. A. R. Quinlan, I. M. Hall, BEDTools: A flexible suite of utilities for comparing genomic features. *Bioinformatics* **26**, 841–842 (2010).

64. S. Anders, P. T. Pyl, W. Huber, HTSeq—A Python framework to work with high-throughput sequencing data. *Bioinformatics* **31**, 166–169 (2015).

65. M. I. Love, W. Huber, S. Anders, Moderated estimation of fold change and dispersion for RNA-seq data with DESeq2. *Genome Biol.* **15**, 550 (2014).

66. S. Kovaka, A. V. Zimin, G. M. Pertea, R. Razaghi, S. L. Salzberg, M. Pertea, Transcriptome assembly from long-read RNA-seq alignments with StringTie2. *Genome Biol.* **20**, 278 (2019).

67. D. W. Huang, B. T. Sherman, R. A. Lempicki, Systematic and integrative analysis of large gene lists using DAVID bioinformatics resources. *Nat. Protoc.* **4**, 44–57 (2009).

68. D. Angeles-Albores, R. Y. N. Lee, J. Chan, P. W. Sternberg, Tissue enrichment analysis for C. elegans genomics. *BMC Bioinformatics* **17**, 366 (2016).

69. J. T. Robinson, H. Thorvaldsdóttir, W. Winckler, M. Guttman, E. S. Lander, G. Getz, J. P. Mesirov, Integrative genomics viewer. *Nat. Biotechnol.* **29**, 24–26 (2011).

70. StataCorp, Stata Statistical Software: Release 14. (2015).

71. J. P. T. Ouyang, A. Folkmann, L. Bernard, C.-Y. Lee, U. Seroussi, A. G. Charlesworth, J. M. Claycomb, G. Seydoux, P granules protect RNA interference genes from silencing by piRNAs. *Dev. Cell* **50**, 716–728.e6 (2019).

72. D. A. H. Nguyen, C. M. Phillips, Arginine methylation promotes siRNA-binding specificity for a spermatogenesis-specific isoform of the Argonaute protein CSR-1. *Nat. Commun.* **12**, 4212 (2021).

73. M. B. Gerstein, Z. J. Lu, E. L. Van Nostrand, C. Cheng, B. I. Arshinoff, T. Liu, K. Y. Yip, R. Robilotto, A. Rechtsteiner, K. Ikegami, P. Alves, A. Chateigner, M. Perry, M. Morris, R. K. Auerbach, X. Feng, J. Leng, A. Vielle, W. Niu, K. Rhrissorrakrai, A. Agarwal, R. P. Alexander, G. Barber, C. M. Brdlik, J. Brennan, J. J. Brouillet, A. Carr, M.-S. Cheung, H. Clawson, S. Contrino, L. O. Dannenberg, A. F. Dernburg, A. Desai, L. Dick, A. C. Dosé, J. Du, T. Egelhofer, S. Ercan, G. Euskirchen, B. Ewing, E. A. Feingold, R. Gassmann, P. J. Good, P. Green, F. Gullier, M. Gutwein, M. S. Guyer, L. Habegger, T. Han, J. G. Henikoff, S. R. Henz, A. Hinrichs, H. Holster, T. Hyman, A. L. Iniguez, J. Janette, M. Jensen, M. Kato, W. J. Kent, E. Kephart, V. Khivansara, E. Khurana, J. K. Kim, P. Kolasinska-Zwierz, E. C. Lai, I. Latorre, A. Leahey, S. Lewis, P. Lloyd, L. Lochovsky, R. F. Lowdon, Y. Lubling, R. Lyne, M. M. Coss, S. D. Mackowiak, M. Mangone, S. M. Kay, D. Mecenas, G. Merrihew, D. M. Miller 3rd, A. Muroyama, J. I. Murray, S.-L. Ooi, H. Pham, T. Phippen, E. A. Preston, N. Rajewsky, G. Rätsch, H. Rosenbaum, J. Rozowsky, K. Rutherford, P. Ruzanov, M. Sarov, R. Sasidharan, A. Sboner, P. Scheid, E. Segal, H. Shin, C. Shou, F. J. Slack, C. Slightam, R. Smith, W. C. Spencer, E. O. Stinson, S. Taing, T. Takasaki, D. Vafeados, K. Voronina, G. Wang, N. L. Washington, C. M. Whittle, B. Wu, K.-K. Yan, G. Zeller, Z. Zha, M. Zhong, X. Zhou, modENCODE Consortium, J. Ahringer, S. Strome, K. C. Gunsalus, G. Micklem, X. S. Liu, V. Reinke, S. K. Kim, L. W. Hillier, S. Henikoff, F. Piano, M. Snyder, L. Stein, J. D. Lieb, R. H. Waterston, Integrative analysis of the *Caenorhabditis elegans* genome by the modENCODE project. *Science* **330**, 1775–1787 (2010).
